# Understanding integrated HPV testing and treatment of pre-cancerous cervical cancer in Burkina Faso, Cote d’Ivoire, Guatemala and Philippines: study protocol

**DOI:** 10.1186/s12978-023-01696-8

**Published:** 2023-11-13

**Authors:** Mark Kabue, Cindy L. Gauvreau, Nemdia Daceney, Margaret Mary Bertram, Tracey Shissler, Veronica Reis, Mathurin Dodo, Ana Garces, Cecilia Llave, Blami Dao, Diwakar Mohan, Lisa Huang

**Affiliations:** 1 Jhpiego, Baltimore, MD USA; 2 The Hospital for Sick Children, Research Institute, Toronto, Canada; 3 Expertise France, Abidjan, Côte d’Ivoire; 4 Jhpiego, Abidjan, Côte d’Ivoire; 5 Jhpiego, Guatemala City, Guatemala; 6 Jhpiego, Manila, Philippines; 7 Jhpiego, Ouagadougou, Burkina Faso; 8 Department of International Health, Johns Hopkins University Bloomberg School of Public Health, Baltimore, MD USA

**Keywords:** Human papilloma virus, Cervical cancer, HIV, Low-and-middle-income countries, Thermal ablation, Burkina Faso, Cote d’Ivoire, Guatemala, Philippines

## Abstract

**Background:**

Many low- and-middle-income countries are disproportionately burdened by cervical cancer, resulting in high morbidity and mortality. HPV-DNA testing coupled with treatment with thermal ablation is a recommended screening and precancer treatment strategy, but not enough is known about how this can be effectively implemented in the context of integrated services. The (Scale Up Cervical Cancer Elimination by Secondary prevention Strategy, (SUCCESS) project is conducting a study to understand this approach, integrated into existing women’s health services in Burkina Faso, Cote d’Ivoire, Guatemala, and the Philippines (2020–2024).

**Methods:**

A hybrid effectiveness-implementation type III mixed-methods observational study design is used to assess feasibility, acceptability, and costs of integrated service delivery in 10 sites per country, selected considering urban/rural location, facility level, onsite/offsite laboratories, and health services type. In each country, a sample size of 2227 women aged 25–49 years will be enrolled with about 20% being women living with HIV. The primary outcome is proportion of HPV positive women completing precancer treatment, if eligible, within three months of screening. Data collection and analysis includes; facility and client exit surveys, key informant and client interviews, registries and project records extractions, and costing data analysis. Analysis includes descriptive statistics, context description, thematic analysis, and document analysis. Quantitative analyses will be stratified by participant’s HIV status.

**Discussion:**

Recruitment of study participants started in April 2022 (Burkina Faso and Côte d’Ivoire) and August 2022 (Guatemala and the Philippines). Enrolment targets for women screened, client exit, in-depth and key informant interviews conducted were reached in Burkina Faso and Cote d’Ivoire in November 2022. Guatemala and Philippines are expected to complete enrolment by June 2023. Follow-up of study Participants 12-months post-treatment is ongoing and is expected to be completed for all countries by August 2024. In LMICs, integrating cervical cancer secondary prevention services into other health services will likely require specific rather than incidental recruitment of women for screening. Reconfiguration of laboratory infrastructure and planning for sample management must be made well in advance to meet induced demand for screening.

*Trail Registration* ClinicalTrials.Gov ID: NCT05133661 (24/11/2021).

**Supplementary Information:**

The online version contains supplementary material available at 10.1186/s12978-023-01696-8.

## Abstract (plain language summary)

Low- and middle-income countries bear a disproportionate burden of cervical cancer deaths and suffering from a disease that is preventable and treatable. Unitaid is funding and working collaboratively with the governments of Burkina Faso, Côte d'Ivoire, Guatemala, and the Philippines to implement Scale Up Cervical Cancer Elimination with Secondary prevention Strategy (SUCCESS) project. Significant advancements on cervical cancer management have been made in last four decades. However, the optimal approaches to translating available evidence into actionable screening and treatment implementation strategies in the context of mass-screening programmes is uncertain. SUCCESS project aims to generate evidence on how to deliver cervical cancer services in a way that promotes human papilloma virus (HPV) screening using newer diagnostic and treatment technologies available. This mixed methods study aims to contribute to the evidence on how HPV testing and treatment can be done in manner that is acceptable and feasible in the context of integrated service delivery. Quantitative and qualitative data will be collected over a period of 24 months (2022–2024) across the four countries. In each country, 2227 women aged 25–49 years will be enrolled and screened for HPV, with 20% of them being women living with HIV. In this study, HPV positive will be retested 12-months after initial screening and/or treatment. The findings of the study will add to the body of literature especially from low- and middle-income countries how to implement promising strategies for scaling up cervical cancer screening and treatment coverage.

**Supplementary Information:**

The online version contains supplementary material available at 10.1186/s12978-023-01696-8.

## Background

Cervical cancer is the fourth most common cancer among women globally. It is the leading cause of death among women living with HIV infection [1–3]. 90% of cervical cancer-related deaths occur in low- and middle-income countries (LMICs), where there is often under-resourced healthcare infrastructure [4]. Based on available evidence over the past decade showing that HPV testing is more sensitive and reliable for the detection of cervical intraepithelial neoplasia compared to cytology and visual inspection with acetic acid (VIA), the WHO recommends using high-performance molecular testing for primary screening [1]. Continued use of VIA for primary screening is therefore not considered to be an effective strategy for scaling up cervical cancer prevention and treatment services especially in LMICs [5–7]. Offering timely cervical cancer secondary prevention measures with new technologies such as HPV test for primary testing and thermal ablation for pre-cancerous lesions treatment, through integration into existing primary health programs may improve access, and provide a platform for population-based screening. Some LMICs like the Philippines [8], having developed national cervical cancer screening programs and others, like Tanzania and Nigeria have costed strategic plans to guide implementation [9]. In resource-limited settings, critically needed services like HPV DNA testing might be expanded through a strategy of integrating them into existing services that are well-established, trusted and related in terms of operations, for example family planning [10, 11]. However, there is limited evidence globally on effective implementation strategies for providing cervical cancer prevention and treatment services at scale as part of integrated health services.

The SUCCESS (Scale Up Cervical Cancer Elimination by Secondary prevention Strategy) project is a Unitaid-funded a consortium led by Expertise France in collaboration with Jhpiego and UICC. The implementation research component of the project is designed to facilitate the generation of evidence regarding the introduction of HPV-DNA testing and pre-cancer treatment through thermal ablation in Burkina Faso, Cote d’Ivoire, Guatemala and the Philippines [12].

The goal of the study is to generate evidence on the feasibility, acceptability, and cost of integrating the HPV plus thermal ablation strategy into the four existing health systems. The study is embedded in the project activities and aims to answer crucial implementation questions identified by the WHO as highly relevant to low-resource settings. SUCCESS thus aligns strategically with the WHO call for the global elimination of cervical cancer in generating information that will contribute towards reaching the 2030 cervical cancer elimination goals.

This paper describes the approach and process employed by SUCCESS project to understand the acceptability and feasibility of providing HPV DNA testing and treatment of pre-cancer cervical lesions with ablative treatment for women 30–49 years in the general population and among women living with HIV (WLHIV) aged 25–49 years as recommended by WHO [13]. The findings of the study will inform the country strategy and guidelines on offering integrated quality cervical cancer secondary prevention services in a manner that is culturally sensitive, client oriented, and system appropriate.

## Methods/design

### Design

**Fig. 1:**
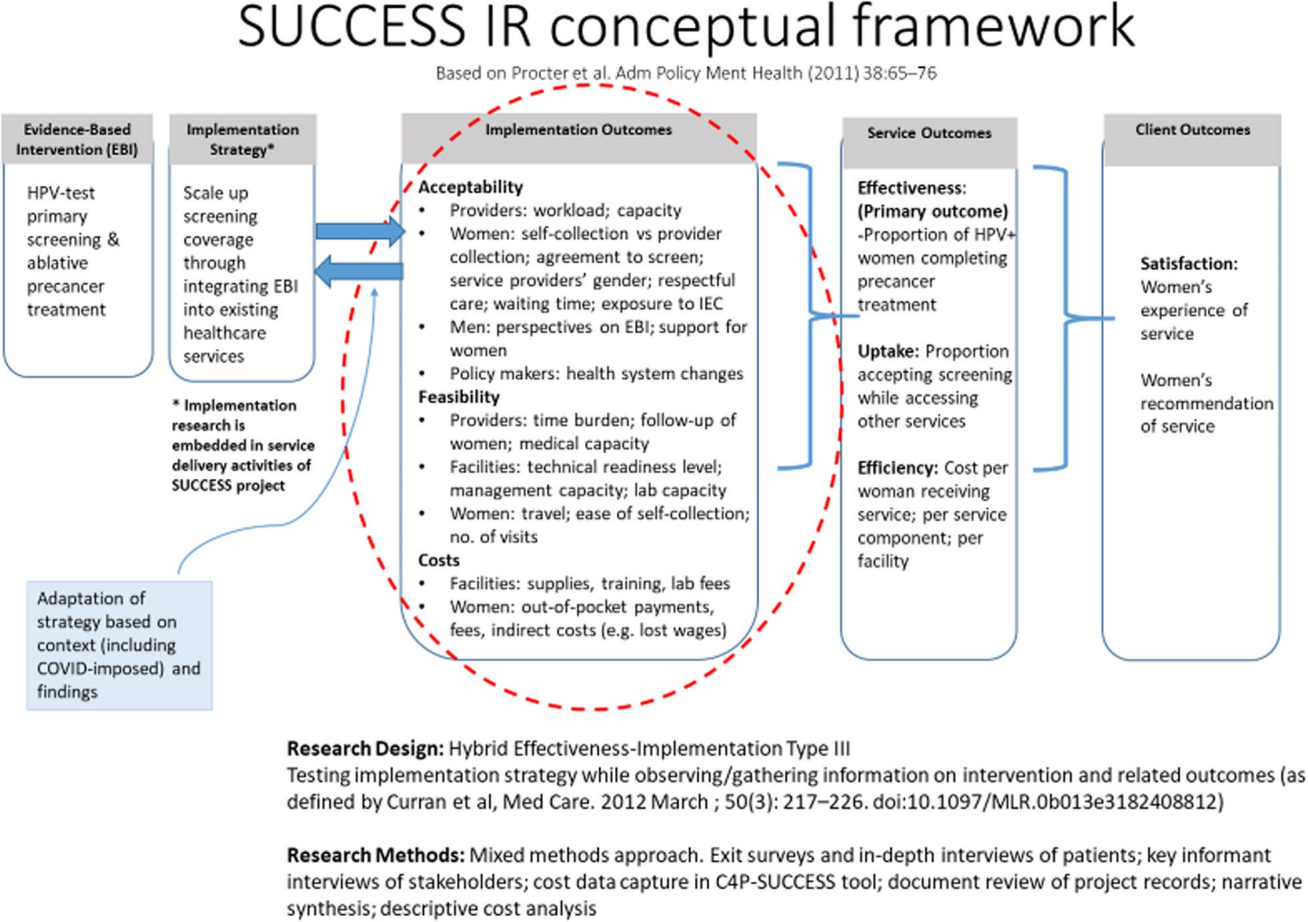
Implementation research conceptual framework for SUCCESS HPV study

This is a hybrid effectiveness implementation Type III study [14], using mixed methods approach for data collection. It’s multi-country, conducted across three continents (Africa, Asia, and Central America), and registered in the ClinicalTrials.gov registry—ID# NCT05133661 (24/11/2021). The implementation research conceptual framework was modelled on the approach described by Proctor et. al, is shown in Fig. 1 [15]. Specifically, we closely observe and study locally relevant contexts and processes, to understand the implementation of services and client outcomes.

The primary outcome is the proportion of HPV positive women (general population and WLHIV) who undergo treatment for precancerous cervical lesions within three months of screening—the optimal time for quality services. Acceptability of services is assessed through measuring satisfaction positive perceptions, and uptake of the cervical cancer services by the users and the service providers. Feasibility of implementation measures the practicability of providing the cervical cancer services through the mechanisms that exist in the four study countries. The unit cost of providing the services as well as user out-of-pocket cost will be also be determined.

### Research questions

The overall research question is: When integrating HPV screening and treatment of pre-cancer lesions with ablative treatments into reproductive health and HIV services, what factors affect the feasibility and acceptability among screen-eligible women and facility-level health care providers in each of the four countries?

Specific research questions are:

1. How feasible is it to implement integrated HPV testing (including self-collection of samples) and ablative treatment of pre-cancerous cervical lesions among HPV positive women aged 30–49 years (general population) and 25–49 years (WLHIV), leveraging on the existing health systems in the four study countries?
2. What is the acceptability of HPV screening through self-collection or clinician collection of sample, and ablative treatment of precancerous lesions among women accessing cervical cancer services, and among service providers in the study sites?
3. What is the cost of implementing integrated HPV screening and cervical precancer treatment services (Supply: counselling, HPV testing, treatment, training health care providers, etc.), and user-related costs (travel, out of pocket expenses opportunity costs, etc.) in the SUCCESS project supported sites?
4. Which factors influence the successful implementation of integrated HPV screening and treatment services (e.g. perceptions, experience of care, religious beliefs, culture, individual characteristics, availability of services, cost, etc.) in the four study countries?

### Setting

In each country, 10 study sites were selected considering a balance of location (e.g. rural/urban), level of services (primary/secondary/tertiary), workload at various service delivery points in a facility (e.g. HIV, postnatal, maternal and new-born health clinic), and the laboratory services available (onsite/ offsite). Where multiple facilities met the criteria, proportionate allocation of the sample was done to achieve balance.

### Participants and sampling

The study relies on multiple approaches to answer the research questions which in turn necessitates the use of different approaches to sampling of the relevant study sub-populations. The four sample components are presented.

Prospective component: In each country, we are enrolling at the time of screening 2,227 women (general population [1787] and WLHIV [440]) and following for 12-months post-treatment those among them who test HPV positive. Assuming an average HPV positivity rate of 15%, we computed that we will follow 268 and 141 from general population and WLHIV, respectively. This will help us to determine the proportion who complete screen-to-treat cycle within three months of screening—the primary outcome of the study. The sample size was computed to a precision of ± 5% and power of 80 percent, design effect of 2 due to clustering at health facilities, and adjusted for loss to follow up at 20%.

Cross-sectional component: To assess the client experience of care and collect some cost data from the user’s perspective, a sample size of 125 women per country was computed assuming that about 1,000 women are available to participate in client exit interview after HPV screening and enrolment, during a 3-month enrolment period (https://www.radar-project.org/isaqoc) [16].

Qualitative data: Fifty in-depth interviews (IDIs) among women who either accept or decline screening/treatment are being conducted in each country. In addition, sixty-eight key informant interviews (KIIs) among service providers, health facility and program managers, policy makers, community mobilizers and men in the community are being conducted. The qualitative data will be triangulated with the quantitative data to help us understand contextual factors that affect implementation of HPV DNA testing and treatment at scale.

Cost data: In addition to the client exit interview cost data, additional data are being collected through time-and-motion observation to document inputs at service delivery (e.g. staff time, supplies, number of procedures per day, laboratory-related processes etc.). Project financial records for 2022–23 will be analysed. The cost database will be maintained using a modified version of the C4P costing tool [17].

### Recruitment and consent

Written informed consent will be obtained from each study participant in accordance with the approved study protocol and documents, prior to data collection. For women recruited in study sites, service providers introduce the study to potential study participants and those who meet the basic criteria (e.g. age, not pregnant, no history of hysterectomy), and are interested in learning more about the study, will be referred to a research assistant for further screening and consenting prior to enrolment. Different consent forms will be used for IDI and KII participants.

### Data collection and timeline

**Fig. 2:**
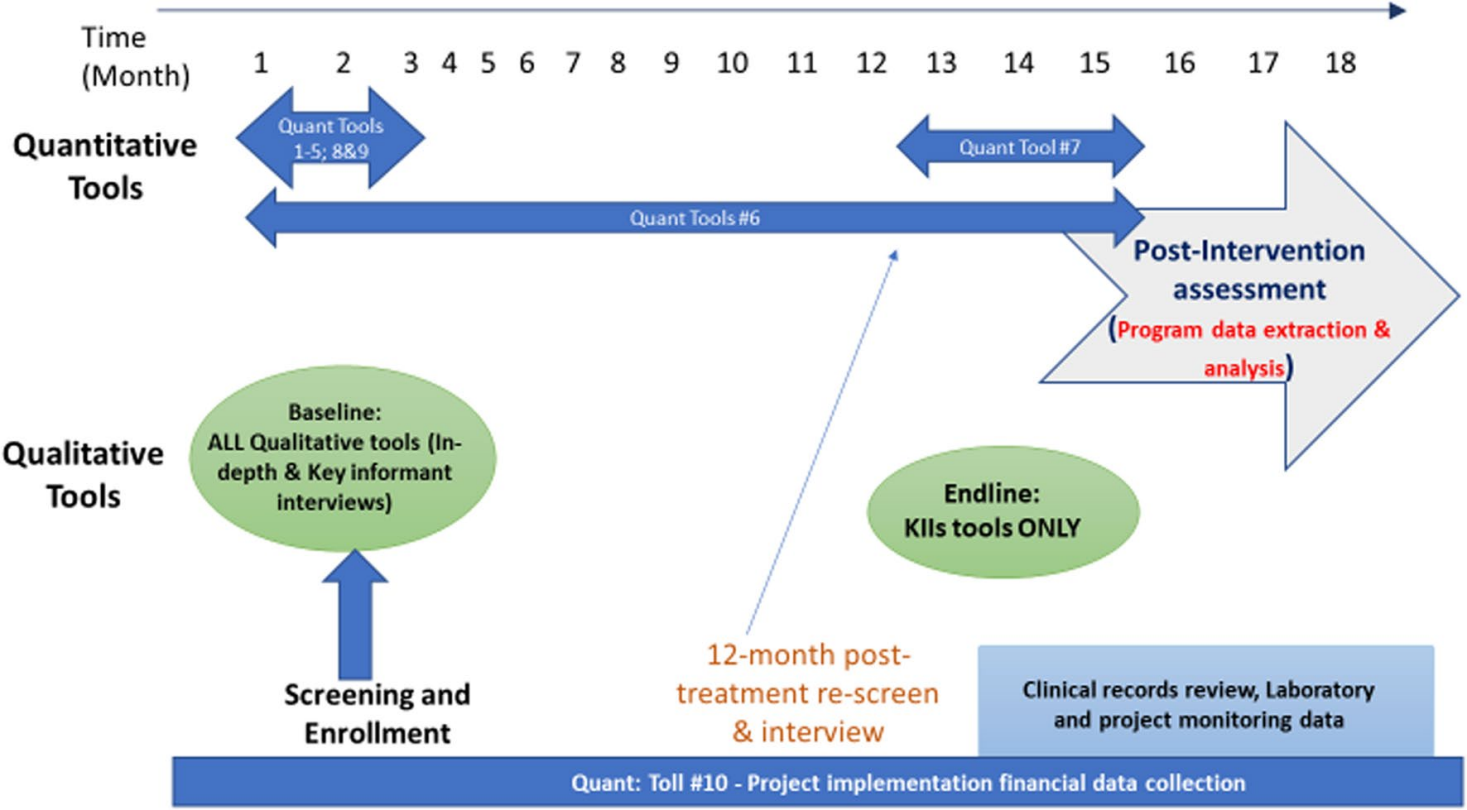
Data collection schedule

The overall duration of the study in each country is estimated to be 18 months, with countries starting and ending at different times depending on when relevant ethical clearance and other preparatory activities are completed. Figure 2 summarizes the data collection schedule through both approaches.

Quantitative data are collected using tablets loaded with the CommCare application (https://www.dimagi.com/commcare/), and uploaded on a central server hosted by Amazon Web Services. This allows for online entry of data in real-time and retrieval. Data are stored in password protected files or on a part of the server where access can be limited to study staff.

Qualitative data are collected by experienced qualitative researchers and audio recorded (audio, MP3 format or equivalent) and note taken. Audio-recorded data files are then being downloaded into to a secure, password protected computer for transcription. Audio recording are stored in locked cabinets until transcription and analysis is complete. Data collection instruments used in the study—quantitative and qualitative—are provided (see Additional file 1, and Additional file 2).

### Research team and governance

**Fig. 3:**
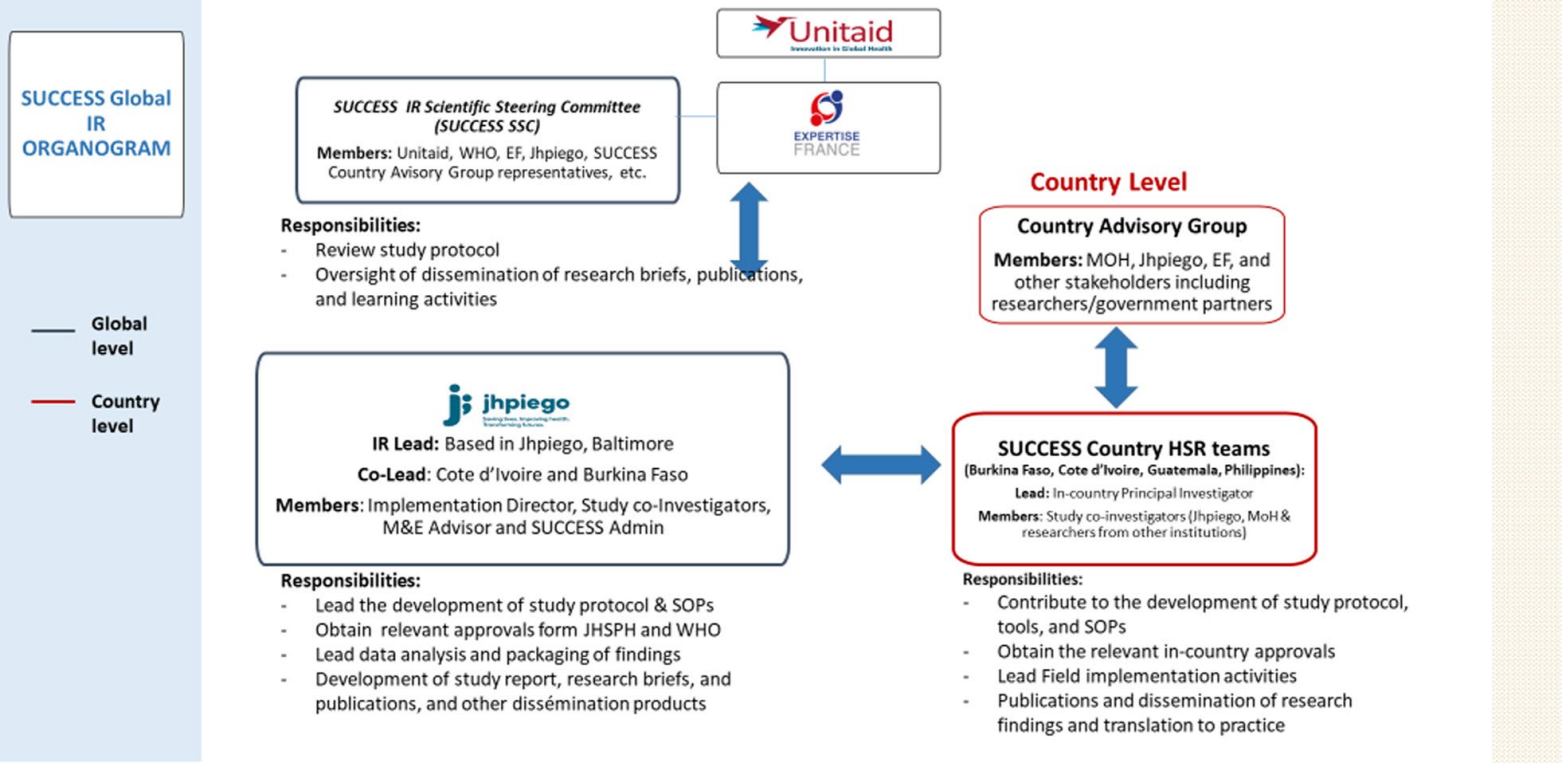
SUCCESS study global level structure

A locally recruited research coordinator and a small team of local data collectors is based in each of the four countries. The principal investigator (PI) is based at Jhpiego headquarters in Baltimore, USA and is providing study implementation oversight across the for countries, including in-person training of the study teams. The PI is supported by a team of globally-based co-investigators, each bringing relevant expertise required to implement the study. Scientific oversight and guidance is provided at the global level by a Study Steering Committee comprising nine members form four National Cancer Institutes globally, and one representative from the four study countries (see Fig. 3).

**Fig. 4:**
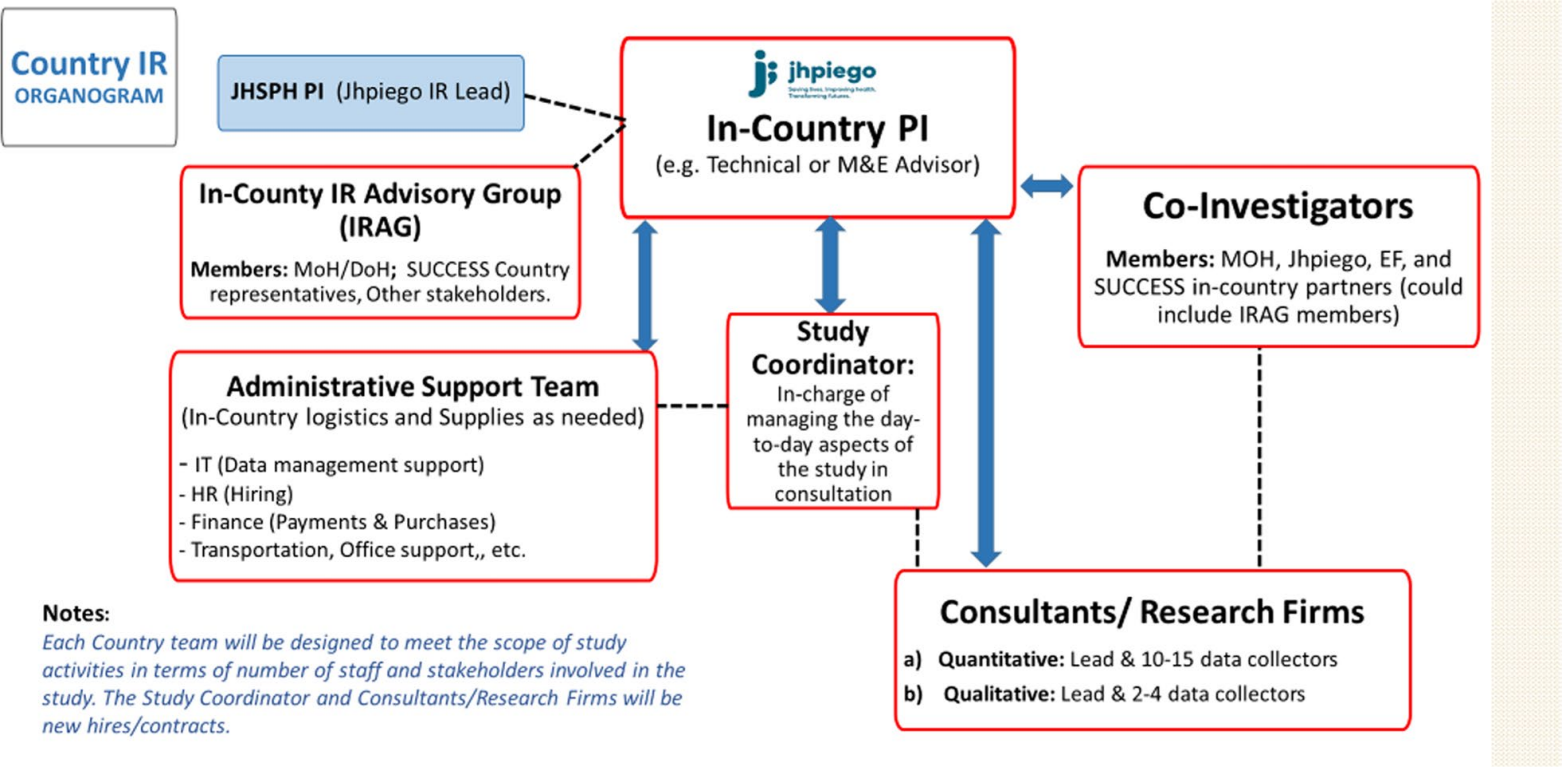
SUCCESS Country level study structure

The lead investigator in each country provides oversight over the study in collaboration with the PI, while the research coordinator is responsible for the day-to-day study activities including supervising the research assistants. Each country study team is working in close consultation with the local Ministry or Department of Health (national and sub-national level) as well an Implementation research advisory group that was formed in each country, in the context of the existing administrative structures (see Fig. 4).

### Monitoring and quality assurance

The study data are available to the study investigators and study coordinators in each country in real time thus facilitating prompt review and correction in a timely manner as needed. Additionally, study coordinators in each country visit each site at least once every four weeks to review verify the data with the source documents in the site. These measures will assure data quality and authenticity. Access to data is restricted to appropriate persons only ensure data safety. Data are stored electronically in a cloud-based system, with an internet protection firewall and the data is backed up weekly. Two-level password authentication is required to access the data by authorized study staff only. Any paper records like logs that may have personal identifiers are stored in a locked cabinet at Jhpiego office in each country.

For qualitative data, the audio recordings are stored in locked cabinets and will be destroyed at the end of the study after verification and data analysis is completed. Only study staff directly involved in collecting, transcribing, or analyzing the qualitative data have access to the recordings. The transcribed electronic versions of the recordings, without personal identifiers, will retained with other study data for 5 years after the end of the study. Consent forms will be kept for the same period to allow respondents to exercise their right of access (Additional file 1).

### Data analysis plan

Descriptive and logistic regression analysis will be conducted for quantitative data to answer the primary and secondary research questions. Additionally, secondary analysis of service delivery data, including laboratory services, will be conducted to determine key parameters such as turnaround time for HPV test results and promptness of treatment where indicated. Cost analysis will determine the unit cost of providing the services as well as user costs. Specifically, cost per woman screened, per woman screened-and-treated, and per delivery component will be reported, by month, year and facility type. Aggregated site-specific, country-specific and project level costs will also be reported. Where comparison is possible to provide insights into cost efficiencies, they will be reported. Anonymized out-of-pocket costs will be consolidated in a separate dataset and also analysed with descriptive statistics (Additional file 2).

KIIs and IDIs data will be transcribed from the audio-recordings and the notes taken, and for recordings in a local language, translated and back translated. NVivo software will be used for analysis. A priori coding framework will be developed for each tool based on existing evidence. These codes will then be applied independently by at least two researchers to three transcripts from each country to refine the coding framework and code definitions, incorporating inductive coding as part of the process. Inter-rater agreement will be used to assess concordance between the researchers. A finalized code book will be applied independently for line-by-line coding to the remaining transcripts for each country. The emerging themes will be iteratively developed by repeatedly analysing data collected from participant categories. Verbatim text generated alongside the code matrix will be used to support the emerging thematic framework. Text and graphical depictions will be used to present summaries. 

### Ethical considerations and approvals

Relevant ethical clearances and approvals were obtained from respective ethical review committees and/or institution review boards as per the research guidelines stipulated for each participating organization. Table 1 shows the institutions that have approved the study. Progress, adverse event, incident and other reports will be submitted to the respective ethics bodies as per the approved study protocol.

**Table 1:** Ethics clearance boards/committees

IRB/REC name	FWA number
WHO Research Ethics Review Committee (Protocol ID: ERC.0003618)	
USA: Johns Hopkins School of Public Health Institution Review Board	FWA00000287
Unitaid: World Health Organization’s Research Ethics Review Committee	FWA00007093
Burkina Faso: Comité d’Ethique de Recherche en Santé du Burkina Faso	FWA00000401
Cote d’Ivoire: Comite National d’Ethique des Sciences de la vie et de la Sante	FWA00025586
Guatemala: Institute of Nutrition of Central American and Panama	FWA000742
Philippines: UP Manila National Institute of Health IRB; and Single Joint Research Ethics Board	FWA00018728

## Discussion

### Study status

All four countries are currently enrolling study participants. Data collection started in April 2022 in Burkina Faso and Cote d’Ivoire, while in Guatemala and the Philippines, data collection started in August 2022. Enrolment targets for women screened, client exit, in-depth interviews and key informant interviews conducted were reached in Burkina Faso and Cote d’Ivoire by end of November 2022. Guatemala and Philippines are expected to complete enrolment by June 2023. The 12-months post-treatment follow-up of women enrolled is expected to be completed for all countries by August 2024.

### Expected outcomes and dissemination of findings

The findings will be disseminated as they become available and used to inform program activities, i.e. continuous learning and adaptation. Overall, the findings of the study will be shared by SUCCESS project for use by the Ministries of Health in the four study countries and other stakeholders to inform the design and implementation of effective strategies of providing cervical cancer services, thus contribute towards reaching the 2030 cervical cancer elimination goals. Wider dissemination will be done nationally and internationally through scientific meetings or conferences as well as publication in peer reviewed journals.

A major output of the implementation research is continuous knowledge translation, both for SUCCESS project improvement and for dissemination to the wider international community that is involved in mitigating the cancer burden in LMICs. We believe that the unique opportunity to conduct research alongside project activities, the wide-reach of the collaboration and the fact that SUCCESS is one of the major vehicles for executing the global initiative to eliminate cervical cancer.

### Supplementary Information

**Additional file 1.** Qualitataive data collection tools.

**Additional file 2.** Quantitative data collection tools.

## Data Availability

Data: not applicable. Supporting/relevant study tools are provided as Additional files.

## Abbreviations

DNA: Deoxyribonucleic acid
DHIS2: District Health Information System-2
FWA: Federal Wide Assurance
HIV: Human immunodeficiency virus
HPV: Human papilloma virus
IDI: In-depth interview
IRB: Institutional Review Board
JH BSPH: Johns Hopkins Bloomberg School of Public Health
KII: Key Informant Interview
LMIC: Low- and Middle-income Countries
PI: Principal Investigator
SUCCESS: Scale Up Cervical Cancer Elimination by Secondary prevention Strategy
VIA: Visual inspection with acetic-acid
WLHIV: Women living with HIV
WHO: World Health Organization
