# Supplementary material for: Understanding integrated HPV testing and treatment of pre-cancerous cervical cancer in Burkina Faso, Cote d’Ivoire, Guatemala and Philippines: study protocol
Source: Reprod Health. 2023 Nov 13;20:167. doi: 10.1186/s12978-023-01696-8 (PMC10644460; doi:10.1186/s12978-023-01696-8)
Supplement: Supplementary file 1 — Additional file 1. Qualitataive data collection tools. [file 12978_2023_1696_MOESM1_ESM.zip › Qualitative tools/10-Key Informant Interview - Program manager and decision makers.docx]

**Study Title:**  Feasibility and Acceptability of HPV testing and Treatment of Precancerous Cervical Lesions in Burkina Faso, Côte d'Ivoire, Guatemala, and Philippines

**Principal Investigator:** Mark Kabue, Dr.PH

**JHSPH IRB No.:** 13630

**PI Version/Date:** v2/ October 15,, 2021

| **Data Collector Number:** |  |
| --- | --- |
| **Interview date:** |  |
| **Participant Study ID:** |  |
| **Number of years of experience in this position:** |  |

***Instructions***

*Please use this form to interview a Health Program managers, Administrators and Policy makers. This interview is designed to gather information about service organization, and the integration and sustainability of the HPV screening at program level.*

*Before beginning the interview, please obtain informed consent from the respondent for their willingness to participate in the study and their permission to audio record the interview using the stamped consent form.*

**Introduction**

1. What is your job title/ position?
2. Please describe your role in your institution / organization.

**Access/ Acceptability / Availability of cervical cancer services**

1. Please describe the availability of cervical cancer screening and treatment services (HPV screening/VIA screen/Pap Smear/Cryotherapy/LEEP/LLETZ) in your area (Specify the area, e.g. district).
2. What is your perception of the HPV screening method compared to the VIA screening method (and Pap smear screening method, if available)?
3. Would you say HPV screening is acceptable to women in your area? Why or why not?
4. Would you say HPV screening followed by visual assessment and treatment for those women who test HPV positive - VAT is acceptable to women in your area? Why or why not?
5. Please describe whether or not HPV done in the home would be acceptable to women in your community from the perspective of your role.
6. What kind of policies or strategies would be most helpful to encourage or support women to do HPV self-collection for screening at home?
7. What are some potential benefits of adopting an approach that integrates HPV screening with VAT? What are the strengths of the HPV self-collection screening approach?

**Barriers and facilitators**

1. What are some potential challenges or obstacles of adopting the HPV screening and VAT approach?
   1. *Probe:* What personal-level or community-level barriers do you think might prevent a woman from accepting HPV screening?
   2. *Probe:* What personal-level or community-level barriers might prevent a woman from going to/returning to the clinic for VAT?
   3. *Probe:* What facility-level barriers might prevent a woman from accepting HPV self-collection for screening?
   4. *Probe:* What facility-level barriers might prevent a woman from going to/returning to the clinic for VAT?
   5. *Probe:* What health system-level barriers might prevent a woman from accepting HPV self-collection for screening?
   6. *Probe:* What health system-level barriers might prevent a woman from going to/returning to the clinic for VAT?
2. How can we facilitate the process that women who screen positive for HPV follow through to receive their results, and treatment?

**Sustainability**

1. How has integration of HPV screening with other programs been done in your area?
   1. *Probe:* What are other implementation considerations, such as within the community?
   2. *Probe:* Can you share any specific features that should be considered when designing community-based HPV screening?
2. How can HPV screening and VAT is an approach be sustained? Could be scaled up?
   1. *Probe:* If more women were doing HPV self-collection, could the lab process more tests?
   2. *Probe:* Could the clinic perform more VATs?
3. Would you recommend HPV screening and VAT to the MOH for scale up? Why or why not?
   1. *Probe:* What are some enablers that you see to the adoption and scale up of HPV testing? Why?
   2. *Probe:* What are some potential barriers that you see to the adoption and scale up of HPV testing? Why?
4. Which screening and treatment approach has the potential to reach the greatest number of women? Why?
5. What improvements can be made to the current screening and treatment approach?
6. Is there anything else you would like to tell me that you did not mention previously?

________________________________________________________________________________________________________________

***thank the Program manager, Administrator or Policy maker for his/her time and participation in the interview.***
