# Supplementary material for: Understanding integrated HPV testing and treatment of pre-cancerous cervical cancer in Burkina Faso, Cote d’Ivoire, Guatemala and Philippines: study protocol
Source: Reprod Health. 2023 Nov 13;20:167. doi: 10.1186/s12978-023-01696-8 (PMC10644460; doi:10.1186/s12978-023-01696-8)
Supplement: Supplementary file 1 — Additional file 1. Qualitataive data collection tools. [file 12978_2023_1696_MOESM1_ESM.zip › Qualitative tools/11-Key Informant Interview - Male community members.docx]

**Study Title:**  Feasibility and acceptability of implementing integrated HPV testing and treatment of pre-cancerous cervical cancer lesions in Burkina Faso,  Côte d'Ivoire, Guatemala, and Philippines

**Principal Investigator:** Mark Kabue, Dr.PH

**JHSPH IRB No.:** 13630

**PI Version/Date:** v2/ October 15, 2021

| **Data Collector Number:** |  |
| --- | --- |
| **Interview date:** |  |
| **Participant Study ID:** |  |
| **Relationship with woman respondent:** |  |
| **Location of interview** |  |

***Instructions***

*Please use this form to interview males who are 18+ years (married or cohabiting with female partners), recruited from the community. This interview is designed to gather information about the perspectives of males towards cervical cancer prevention, especially screening through HPV screening and treatment in health facilities and in the community. If feasible and acceptable, we will interview spouses of some women who are enrolled in the study (accepted HPV screening and/or treatment).*

*Before beginning the interview, ascertain the age (Must be 18+ years), obtain informed consent from the respondent for their willingness to participate in the study and their permission to audio record the interview using the stamped consent form.*

**Introduction**

1. How old were you during the last birthday?
2. What do you do for a living? What is the main source of your income?
3. Have you heard of cervical cancer? If yes, what do you know about it?
   1. **Note:** If participant does not know about cervical cancer; provide a brief description and use it as a basis for the questions that follow (Scenario-based).
   2. **Note:** If participants knows about cervical cancer, base the questions that follow on what he knows as a reference point.
4. Please describe your understanding of the risk of cervical cancer to women in general.
   1. *Probe*: How serious a problem is it? How widespread is it? Know or heard of anyone who died from it?

***Cervical cancer prevention and treatment***

Some women in this community may accept to be screened for cervical cancer while other may not agree *(Briefly describe “Self-collection” and the “Clinician collection” of sample procedures to the respondent*).

1. What could make women want to be screened through either using self-collection or clinician collecting the sample?
2. *Probe:*Ask for reasons/concerns such as privacy, convenience, and capacity/skill to perform the task.
3. What could make women NOT be willing to be screened for cervical cancer through either self-collection or Clinician collection of sample?
4. *Probe: Ask for* reasons that might make a woman not want to be screened, e.g. cost, time, inadequate information, etc.
5. What could be done to make it easier for women to go to a health facility for screening and treatment for cervical cancer?
6. Probe: e.g. providing financial support to women, etc.

**Social support**

1. Do women in this community go to health facilities on their own to seek health services? Do they usually need permission from their husbands or partners to go to the health facility? What about other family members like mother in-law, uncle, etc.? Explain.
2. Have you discussed with your wife or partner about cervical cancer? Would you be willing to have this discussion? Why? Why not? Explain.
3. Would you accompany your wife to the clinic for screening and/or treatment for cervical cancer? If no, why not? If yes, what’s the motivation?
4. If you had (or have) a daughter, would you be willing to discuss with her about cervical cancer? If yes, at what age? Explain.
5. What challenges are you aware of regarding what is being done to prevention and treatment of cervical cancer?
6. Probe: How can these challenges be addressed? What role can men play? e.g. learning more about diseases affecting women, etc.
7. Is there anything else you would like to tell me that you did not mention previously?

***thank the MALE RESPODENT for his time and participation in the interview.***
