# Supplementary material for: Understanding integrated HPV testing and treatment of pre-cancerous cervical cancer in Burkina Faso, Cote d’Ivoire, Guatemala and Philippines: study protocol
Source: Reprod Health. 2023 Nov 13;20:167. doi: 10.1186/s12978-023-01696-8 (PMC10644460; doi:10.1186/s12978-023-01696-8)
Supplement: Supplementary file 1 — Additional file 1. Qualitataive data collection tools. [file 12978_2023_1696_MOESM1_ESM.zip › Qualitative tools/1-Indepth interview - Women who Accept HPV Screening.docx]

**Study Title:** Feasibility and acceptability of implementing integrated HPV testing and treatment of pre-cancerous cervical cancer lesions in Burkina Faso,  Côte d'Ivoire, Guatemala, and Philippines

**Principal Investigator:** Mark Kabue, Dr.PH **JHSPH IRB No.:** 13630 **PI Version/Date:** v2/ October 15, 2021

| **Data Collector Number:** |  |
| --- | --- |
| **Facility Study ID:** |  |
| **Today’s date:** |  |
| **Participant Study ID:** |  |
| **Date of HPV Sample Self-Collection:** |  |
| **Date of HPV Clinician Sample Collection:** |  |

***Instructions***

*Please use this form to interview Women who accept HPV Screening though either self-collection or clinician-collection of sample. This interview is designed to gather information about organization of cervical cancer services, the clients’ experience of care and views of acceptability of the Self-Collection as well as clinician collection of sample for HPV screening.*

*Before beginning the interview, please obtain informed consent from the respondent for their willingness to participate in the study and their permission to audio record the interview using the stamped consent form.*

**Introduction Questions (Both clinician and self-collection)**

1. How old were you during your last birthday?
2. Do you have any children? How many? How old are they?

**Awareness (Both clinician and self-collection)**

1. Prior to coming to this facility, had you ever heard of cervical cancer?
2. What had you heard or believed causes cervical cancer? Had you heard of human papilloma virus or HPV?
3. How concerned are you about developing cervical cancer?
4. What are your main sources of information on cervical cancer?
5. *Probe:* If one source like radio is mentioned, ask “which other source” to seek more responses. Do not mention any source. Only prompt for more responses. Have you been previously screened for cervical cancer? If yes, when? [Note the year and month if known]. What was the result and what was done after that? [Document if known]
6. Please let me know your preference: clinician collection of sample or self-sampling?
   1. If self-sample, would you prefer doing it at home or in a health facility? Explain your choice, please.

**Experience/Screening Choice (Self-collection ONLY):** I am trying to better understand what women’s preferences are for screening methods, what is acceptable to women in this community, and why. I would really like to hear your perspective on your experience with HPV self-collection, where you collected your own sample for testing with the swab or soft brush.

1. Please describe your personal experience of self-collection of the sample and where it was performed.
2. Why did you choose (or agree to) self-collection of the sample?
3. What did you like about the self-collection? What do you think are benefits of doing self-collection?
4. What did you dislike about self-collection? What are possible challenges?
5. How did you feel before you used the self-collection kit?
   1. *Probe:* What kind of emotions were you experiencing?
6. If you had the option, would you choose to do self-collection at home or at a health facility? Why?
   1. *Probe:* What are the benefits for doing self-collection in your home? What are the challenges?
   2. *Probe:* What are the benefits for doing self-collection in the health facility? What are the challenges?
   3. *Probe:* What kind of concerns did you have ahead of time?

**Acceptability/Feasibility questions(Self-collection ONLY):**

[**Interviewer reads]**: Some women may not feel that self-collection is acceptable among the women living in your community].

1. Why would some women not want to be screened using self-collection?
   1. *Probe:* Reasons/concerns that might discourage a woman from self-collection, such as spousal consent.
2. What could make women more willing to accept self-collection?
   1. *Probe:* Reasons that might encourage a woman to prefer to do self-collection.
3. How much do you trust doing the self-collection to give accurate information about your risk for cervical cancer?
4. Assuming that self-collection and collection of the sample by a nurse are equally good at getting accurate test results, which would you prefer for future screening?
5. Would you recommend the HPV self-collection screening method to other women? Why or why not?

**Instructions and Use (Self-collection ONLY):**

1. After reading the instructions (or having the instructions explained to you), please describe your confidence in being able to use the self-collection correctly? Are the instructions clear?
2. Was the self-collection easy to do? Or did you have some difficulty?
3. What was the most difficult part, if any, about using the self-collection?
4. Did you have any physical discomfort during the self-collection? If yes, please describe.
5. How much discomfort did you have doing the self-collection?
6. If given a choice to do self-collection, where would you prefer to do it?

**Results Delivery (Both clinician and self-collection)**

1. Which method of communication do prefer be used to receive your test results? Why?
2. If you didn’t understand your results, what would you do to get more information?
3. In your own words, please describe the next step for screening and treatment approach.
   1. *Probe:* If your results are HPV negative, what will you do?
   2. *Probe:* If your results are HPV positive, what will you do?

**Wrap up (Both clinician and self-collection) of sample**

1. Please share any improvements that could be made to HPV screening process.
2. Is there anything else you would like to tell me that you did not mention previously?

**THANK THE CLIENT FOR HER TIME AND PARTICIPATION IN THE INTERVIEW.**
