# Supplementary material for: Understanding integrated HPV testing and treatment of pre-cancerous cervical cancer in Burkina Faso, Cote d’Ivoire, Guatemala and Philippines: study protocol
Source: Reprod Health. 2023 Nov 13;20:167. doi: 10.1186/s12978-023-01696-8 (PMC10644460; doi:10.1186/s12978-023-01696-8)
Supplement: Supplementary file 1 — Additional file 1. Qualitataive data collection tools. [file 12978_2023_1696_MOESM1_ESM.zip › Qualitative tools/2-Indepth Interview - HPV positive Women who Return for VAT.docx]

**Study Title:** Feasibility and acceptability of implementing integrated HPV testing and treatment of pre-cancerous cervical cancer lesions in Burkina Faso,  Côte d'Ivoire, Guatemala, and Philippines

**Principal Investigator:** Mark Kabue, Dr.PH **JHSPH IRB No.:** 13630 **PI Version/Date:** v2/ October 15, 2021

| **Data Collector Number:** |  |
| --- | --- |
| **Facility Study ID:** |  |
| **Interview date:** |  |
| **Participant Study ID:** |  |
| **Date of HPV Screen:** |  |

***Instructions***

*Please use this form to interview Women who are HPV positive and returned for visual inspection with acetic acid and treatment (VAT). This interview is designed to gather information about service organization, the woman’s experience and her views of acceptability of the HPV screening (Self-collection or Clinician collection of sample), and VAT.*

*Before beginning the interview, please obtain informed consent from the respondent for their willingness to participate in the study and their permission to audio record the interview using the stamped consent form.*

**Introduction Questions**

1. How old were you during the last birthday?
2. Do you have any children? If yes, how old are they?

**Delivery of HPV test results**

1. Please describe how you received your HPV test results.
   1. *Probe:* Explore the woman’s experience and satisfaction with the way the results were delivered to her.
   2. *Probe:* If done in-person, perception whether the space or mode of communication was private enough for the woman.
   3. *Probe:* How the provider communicated the results. What liked or did not like about the interaction with the clinician.
   4. *Probe:* Given a choice, what the preferred way to receive HPV test results would be, and why.
2. Tell me how well you understood your test results when you received them.
   1. *Probe:* Did you understand what your results meant?
   2. *Probe:* Did you need help to understand your results?
   3. *Probe:* If you didn’t understand your results, what did you do, if anything, to get more information?
   4. *Probe:* Who, if anyone, helped you understand what those results meant?
3. Tell me how long it took to receive your results after the sample was collected.
   1. *Probe:* Did you think this was an appropriate or acceptable amount of time?
   2. *Probe:* What is a reasonable time to wait for test results, too long, or too short?
4. Describe how receiving your test results made you feel.
   1. *Probe:* Were you anxious? Sense of relief? Confused?
   2. *Probe:* What thought came to your mind immediately after you were informed of HPV positive results?

**Returning to Health Facility for VAT**

1. Please describe your experience with the visit today for further evaluation and treatment.
2. What made it possible for you to return to the clinic for today’s visit?
   1. *Probe*: Had money for transportation? Had a companion? Spouse or partner gave permission? [Specify the relationship with companion, if women has one].
   2. *Probe*: Received a call from clinician / health facility to come for results?
   3. *Probe*: Received an SMS?
3. Please describe the process for getting or arranging an appointment for further evaluation and treatment of your HPV results.
   1. *Probe:* How could that process be improved?
   2. *Probe:* How easy or difficult is it to get to the facility for evaluation and treatment?
4. What makes it difficult to get to the health facility for further evaluation of your results, where the provider examines your cervix and determines the best treatment for you?
   1. *Probe:* What are some reasons why a woman may not come to complete this type of visit and treatment?
5. Would you say any of your friends or family discourage you from coming for the visit and treatment?
   1. *Probe:* If so, who? What is their relationship to you?
   2. *Probe:* Did you need your spouse’s or partner’s approval/ permission to come for treatment? Ask is partner/ spouse is aware that the woman has to abstain from sex for about four weeks after treatment.
   3. *Probe:* What did they say to discourage you from returning?
6. Briefly describe what could help to make it easier for you, or for other women, to come to the health facility for further evaluation and treatment?
7. Please describe what you think your risk is of developing cervical cancer. Why do you say so?

**Wrap up**

1. Please describe any improvements that could be made to HPV screening and treatment for women who test HPV positive in this community.
2. Is there anything else you would like to tell me that you did not mention previously?

**THANK THE CLIENT FOR HER TIME AND PARTICIPATION IN THE INTERVIEW.**
