# Supplementary material for: Understanding integrated HPV testing and treatment of pre-cancerous cervical cancer in Burkina Faso, Cote d’Ivoire, Guatemala and Philippines: study protocol
Source: Reprod Health. 2023 Nov 13;20:167. doi: 10.1186/s12978-023-01696-8 (PMC10644460; doi:10.1186/s12978-023-01696-8)
Supplement: Supplementary file 1 — Additional file 1. Qualitataive data collection tools. [file 12978_2023_1696_MOESM1_ESM.zip › Qualitative tools/3-Indepth Interview - Women who DECLINE HPV Screening.docx]

**Study Title:**  Feasibility and acceptability of implementing integrated HPV testing and treatment of pre-cancerous cervical cancer lesions with thermal ablation in Burkina Faso,

Côte d'Ivoire, Guatemala, and Philippines

**Principal Investigator:** Mark Kabue, MPH, Dr.PH

**JHSPH IRB No.:** 13630

**PI Version/Date:** v2/ October 15, 2021

***Instructions***

*Please use this exit survey to ask questions of* ***women who are eligible for HPV screening, but decline****. This survey is designed to gather information about the acceptability HPV self-sampling or clinician collection of sample, in the health facility.*

*Ensure that oral consent is obtained from the woman.*

| **Data Collector ID Number:** | (pre-populated) |
| --- | --- |
| **Facility Study ID:** | (pre-populated) |
| **Interview date** | (pre-populated) |
| **Participant Study ID:** | (pre-populated) |

***Instructions: Ask BOTH those who decline either clinician or self-collection of sample***

**Introduction Questions**

1. How old were you during your last birthday?
2. Do you have any children? How many? How old are they?
3. What is the MAIN reason for coming to the facility today?
   1. *Probe*: Services that women was seeking, e.g. FP, TB, HIV, lab test, follow-up, etc.

**Knowledge on cervical cancer, screening, and perceived risk**

1. Before today, had you heard of cervical cancer before? What about human papilloma virus?
   1. *Probe*: If Yes, describe what you know about cervical cancer. What was the source of this information?
   2. *Probe*: As about specific sources of information if none are mentioned voluntarily; e.g. Nurse / midwife; Doctor/Medical Officer; Community health worker; Radio; School; Television; Newspaper; Village Meeting, etc.
2. Have you ever been screened for cervical cancer before?
   1. *Probe*: If Yes, ask when was it done (approx. year and month). Ask what was done after screening.
3. Do you consider yourself at risk of cervical cancer? Explain your answer.

**Refusal of self-collection of sample**

**[Instructions: Ask women who were offered self-collection of the sample and declined]**

1. We understand that there are many reasons why women may not be able or not interested in doing self-collection of sample for HPV testing. What is the reason (or reasons)that you did not test for cervical cancer through collecting?
   1. *Probe:* Privacy concerns? Concerns about the process?
   2. *Probe:* Need to consult partner/ spouse first?
2. If you were to collect your own sample, would you prefer to do it at home or at a health facility? Why?

**Refusal of clinician-collection of sample**

**[Instructions: Ask women who were offered clinician collection of the sample and declined]**

1. We know there are many reasons why women may not be able or not interested in having a clinician collect a vaginal sample from them. What is the reason that you did not agree to a clinician screening you for cervical cancer today?
   1. *Probe*: Need to consult partner/ spouse first?
   2. *Probe*: Would the sex of the clinician matter to you for this process?
   3. *Probe*: If yes, what would be your preference? Male or female clinician?

**Wrap up**

10 . Would you be willing to be screened for cervical cancer at this or another health facility in future?

1. *Probe*: If yes, what would make you agree to be screened?
2. *Probe*: What changes would you suggest be implemented to the cervical cancer screening?

Anything else you would like to share with us?

**THANK THE CLIENT FOR HER TIME AND PARTICIPATION IN THE INTERVIEW.**
