# Supplementary material for: Understanding integrated HPV testing and treatment of pre-cancerous cervical cancer in Burkina Faso, Cote d’Ivoire, Guatemala and Philippines: study protocol
Source: Reprod Health. 2023 Nov 13;20:167. doi: 10.1186/s12978-023-01696-8 (PMC10644460; doi:10.1186/s12978-023-01696-8)
Supplement: Supplementary file 1 — Additional file 1. Qualitataive data collection tools. [file 12978_2023_1696_MOESM1_ESM.zip › Qualitative tools/4-Interview - HPV positive women who do NOT return for VAT.docx]

**Study Title:**  Feasibility and Acceptability of HPV testing and Treatment of Precancerous Cervical Lesions in Burkina Faso, Côte d'Ivoire, Guatemala, and Philippines

**Principal Investigator:** Mark Kabue, Dr.PH

**JHSPH IRB No.:** 13630

**PI Version/Date:**v2/ October 15, 2021

| **Data Collector Number:** | *(pre-populate from Enrolment form)* |
| --- | --- |
| **Facility Study ID:** | *(pre-populate from Enrolment form)* |
| **Client Unique ID:** | *(pre-populate from Enrolment form)* |
| **Date of screening:** | *(pre-populate from Enrolment form)* |
| **Date of HPV test results:** | *(pre-populate from Enrolment form)* |
| **Date of interview:** |  |

***Instructions***

*Use this tool to collect information (Via phone or in-person) from women who are HPV Positive but did not return for VAT within three months after HPV screening. This tool is designed to gather information about the acceptability of the HPV screening, delivery of test results, and subsequent further evaluation and treatment as needed.*

**Introductions and verification of identity**

[**Instructions**:– *Interviewer asks the respondent to confirm her identity by asking the a question whose answer was recorded at enrollment]*

| **Question** | **Response/Codes** |
| --- | --- |
| *If contacted via phone, retrieve SECRET code word recorded at Enrollment to verify identity (e.g. Name of maternal grandmother)* | *(pre-populate from Enrolment form: SECRET code word)* |

1. How old were you during your last birthday?
2. Do you have any children? If so, how old are they now?

**HPV test and receiving results**

1. Did you receive your HPV test results? Briefly describe what happened. [Ask ONLY those who answer “YES”]
   1. *Probe:* How were the results delivered to you?
   2. *Probe:* How were the results communicated? Were you comfortable with the way your HPV test results were delivered? Was the environment private enough?
   3. *Probe:* How did the test results make you feel? Sad? Anxious? Relieved?
2. If you did NOT receive your test results, what could have happened to cause this?
   1. *Probe*: For example, missed a scheduled appointment, moved to another location, etc.

**Visual Assessment and Treatment Follow Up**

1. I would like to you to share with me the challenges that you faced which made it difficult for you to go to the health facility for further evaluation and possibly receive treatment if necessary.
   1. *Probe****:*** Reasons for not returning to the health facility for further assessment and treatment such as not receiving the test results, lack of transport money, spousal permission denied, no time, etc.
   2. *Probe*: Influence of friends or relatives who discouraged you from returning to the clinic, etc.

**Information on cervical cancer and it’s management**

1. What is /are your main source(s) of information on cervical cancer?
   1. *Probe*: As about specific sources of information if none are mentioned voluntarily; e.g. Nurse / midwife; Doctor/Medical Officer; Community health worker; Radio; School; Television; Newspaper; Village Meeting, etc.
2. Would you consider visual assessment and treatment procedure as safe or unsafe? Explain your answer?

**Wrap up**

1. Would you now consider going for visual assessment and treatment in the future?
   1. *Probe*: If Yes, would you go to the same facility where you were tested? Why? Why not?
   2. *Probe*: If Yes, would you go to another facility? Why?
   3. *Probe*: What would need to change for you to go for follow up visit?
2. Is there anything else you would like to share with us?

**THANK THE CLIENT FOR HER TIME AND PARTICIPATION IN THE INTERVIEW.**
