# Supplementary material for: Understanding integrated HPV testing and treatment of pre-cancerous cervical cancer in Burkina Faso, Cote d’Ivoire, Guatemala and Philippines: study protocol
Source: Reprod Health. 2023 Nov 13;20:167. doi: 10.1186/s12978-023-01696-8 (PMC10644460; doi:10.1186/s12978-023-01696-8)
Supplement: Supplementary file 1 — Additional file 1. Qualitataive data collection tools. [file 12978_2023_1696_MOESM1_ESM.zip › Qualitative tools/5-Key Informant Interview - LEEP_LLETZ service providers (Doctors).docx]

**Study Title:** Feasibility and acceptability of implementing integrated HPV testing and treatment of pre-cancerous cervical cancer lesions in Burkina Faso,  Côte d'Ivoire, Guatemala, and Philippines

**Principal Investigator:** Mark Kabue, Dr.PH **JHSPH IRB No.:** 13630 **PI Version/Date:** v2/ December 15, 2021

| **Data Collector Number:** |  |
| --- | --- |
| **Interview date:** |  |
| **Participant Study ID:** |  |
| **Client volume in last month (VIA or VAT):** |  |
| **Number of years of experience in providing LEEP/LLETZ:** |  |
| **Number of years of experience since graduation as a Medical Officer/Doctor:** |  |

***Instructions***

*Please use this form to interview a LEEP/LLETZ Doctor. This interview is designed to gather information about service organization, and the integration of the HPV screening, and provision of LEEP/LLEETZ.*

*Before beginning the interview, please obtain informed consent from the respondent for their willingness to participate in the study and their permission to audio record the interview using the stamped consent form.*

1. How long have you been a doctor at this facility?
2. When were you trained in LEEP/LLETZ
3. How long have you been providing LEEP/LLETZ at this facility and elsewhere?
4. When do you provide LEEP/LLETZ (e.g. days of the week)?
   1. *Probe:* How many days per month?
5. What other clinical services do you provide along with these services?

***Evolving Workload***

1. Previously, women were being referred to LEEP/LLETZ from two streams of screening, pap smear or See and Treat (VIA). From your perspective as a provider, how has the transition from VIA See-and-Treat to HPV screening method affected your work?
   1. *Probe:* What about volume of services provided?
2. How has the transition from VIA See-and-Treat to HPV self-collection and triage with VAT is going? Please describe whether this transition has been helpful or harmful.
3. How has the change from screening with VIA to HPV self-collection had an impact on your workload?
   1. *Probe:* Has the number of women being referred to the LEEP/LLETZ clinic changed? If yes, how?
   2. *Probe:* Have the referral processes changed under this study protocol? If yes, how?
   3. *Probe:* How does that affect your work?
4. Please describe whether or not this change to using HPV testing for screening is likely to be sustainable.
   1. *Probe*: Additional workload burden? Staff skillset needed?
   2. *Probe*: Faster or slower way of assessing clients and offering treatment?
5. In what ways has the switch from VIA See-and-Treat to HPV and VAT affected the quality of services provided at the clinic?
   1. *Probe*: Accuracy of the results? Confidence in diagnosis and recommended treatment?
6. Please describe any improvements that could be made to the current screening and treatment approach.
7. Is there anything else you would like to tell me that you did not mention previously?

***thank the LEEP/LLETZ provider for his/her time and participation in the interview.***
