# Supplementary material for: Understanding integrated HPV testing and treatment of pre-cancerous cervical cancer in Burkina Faso, Cote d’Ivoire, Guatemala and Philippines: study protocol
Source: Reprod Health. 2023 Nov 13;20:167. doi: 10.1186/s12978-023-01696-8 (PMC10644460; doi:10.1186/s12978-023-01696-8)
Supplement: Supplementary file 1 — Additional file 1. Qualitataive data collection tools. [file 12978_2023_1696_MOESM1_ESM.zip › Qualitative tools/6-Key Informant Interview - Service provider.docx]

**Study Title:** Feasibility and acceptability of implementing integrated HPV testing and treatment of pre-cancerous cervical cancer lesions in Burkina Faso,  Côte d'Ivoire, Guatemala, and Philippines

**Principal Investigator:** Mark Kabue, Dr.PH **JHSPH IRB No.:** 13630 **PI Version/Date:** v2/ October 15, 2021

| **Data Collector Number:** |  |
| --- | --- |
| **Interview date:** |  |
| **Participant Study ID:** |  |
| **Client volume in last month (VIA or VAT):** |  |
| **Number of years of experience in providing VIA or VAT:** |  |
| **Number of years of experience since graduation as nurse:** |  |

***Instructions***

*Please use this form to interview service providers in the target units where HPV screening and treatment is provided. This interview is designed to gather information about service organization, acceptability of and feasibility of integrating HPV screening and treatment of precancerous lesions with other services at the health facility level.*

*Before beginning the interview, please obtain informed consent from the respondent for their willingness to participate in the study and their permission to audio record the interview using the stamped consent form.*

***Introductory Questions/Rapport Building***

1. What is your job title?
2. How long have you been serving in that capacity at this facility?
3. Please describe your role at this facility.
   1. *Probe:* Please describe how a typical work day looks like.
4. Have you been on trained in VIA and cryotherapy? What about VIA and thermal ablation? If yes, when was this done?

***HPV Sample self-collection by women***

1. Which sample collection method (HPV Self-Collection or provider collection) do most women seem to prefer?
   1. *Probe:* Do women have a choice on the which of the two methods is used? How is the choice of methods presented to the women?
   2. *Probe:* Why is the method you mentioned most preferred by the women?
2. Are there some women who refuse HPV self-collection at a health facility if its offered to them? What the some of the reasons why they refuse?
3. Why do some women refuse or do not want clinicians to collect samples from them for cervical cancer screening?
   1. *Probe*: Ask about fear, misconceptions of cancer risk or treatment, lack of family support, economic hardships, etc.)
4. As a service provider, how is HPV testing and treatment being implemented at this health facility?
   1. Probe: Is transition to HPV testing and treatment with thermal ablation in this facility changing the way services are provided? If yes, how?
   2. Probe: What has this change meant for you (in terms of workload and service delivery)? What has this change meant for clients (in terms of quality of services)?
   3. *Probe:* What is going well? What are the challenges?
   4. *Probe:* What are the reasons why the Ministry is promoting HPV self-collection?
   5. *Probe:* What do you think of this approach, compared to how cervical cancer screening was done previously?

***Communication/Scheduling visits for clients***

1. What communication channels are being used to follow-up with HPV-positive women to schedule VAT?
2. *Probe:* Who contacts the woman?
3. *Probe:* How many contact attempts does it usually take before you reach them?
4. *Probe:* If a woman does not return your phone calls/contact attempts, what do you do?
5. *Probe:* Please describe another communication method that you think would be more effective than what is currently used.
6. If a woman does not come for her VAT appointment, what do you do?
7. *Probe:* How successful are you at the approach you have described?
8. *Probe:* Does this differ from the procedures in place for scheduling women who postponed cryotherapy following a VIA positive result?

***For Women with HPV positive test results***

1. What are the facilitators or conditions that encourage a woman to come to a health facility for treatment after screening HPV-positive?
   1. *Probe*: What are the strengths of the HPV screening and VAT approach?
2. What are the barriers or challenges that might prevent a woman from coming to a health facility for VAT after screening HPV-positive?
3. *Probe:* Challenges or obstacles of the HPV screening and VAT.

***Workload***

1. How has or would change from screening with VIA to HPV Self-Collection had an impact on your workload as a service provider? If yes, how? What about in this facility in general?
2. *Probe:* Has it changed the number of women that you’re treating with ________ [*Name the treatment modalities offered at the facility*] increased or decreased? What about the number you are referring for LLETZ or suspect cancer?
3. *Probe:* How does that affect how you use your time?
4. How does the change in workload and treatment approach influenced the quality of services provided at this facility?

**Improvements to the Program**

1. Please describe any improvements that could be made to HPV screening and VAT approach.
2. Is there anything else you would like to tell me that you did not mention previously?

***thank the health care provider for his/her time and participation in the interview.***
