# Supplementary material for: Understanding integrated HPV testing and treatment of pre-cancerous cervical cancer in Burkina Faso, Cote d’Ivoire, Guatemala and Philippines: study protocol
Source: Reprod Health. 2023 Nov 13;20:167. doi: 10.1186/s12978-023-01696-8 (PMC10644460; doi:10.1186/s12978-023-01696-8)
Supplement: Supplementary file 1 — Additional file 1. Qualitataive data collection tools. [file 12978_2023_1696_MOESM1_ESM.zip › Qualitative tools/7-Key Informant Interview - Facility or Unit In-Charge.docx]

**Study Title:** Feasibility and acceptability of implementing integrated HPV testing and treatment of pre-cancerous cervical cancer lesions in Burkina Faso,  Côte d'Ivoire, Guatemala, and Philippines

**Principal Investigator:** Mark Kabue, Dr.PH **JHSPH IRB No.:** 13630 **PI Version/Date:** v2/ October 15, 2021

| **Research Assistant code:** |  |
| --- | --- |
| **Date of interview:** |  |
| **Participant ID:** |  |
| **Participant’s position (e.g. Chief Doctor/ Nurse):** |  |
| **Number of years of experience in this position:** |  |
| **Client volume in last month (VIA or VAT):** |  |

***Instructions***

*Please use this form to interview Facility or Unit In-Charge. This interview is designed to gather information about service organization, integration of services and sustainability of HPV screening nd treatment in the facility.*

*Before beginning the interview, please obtain informed consent from the respondent for their willingness to participate in the study and their permission to audio record the interview.*

**Background/Warm Up**

1. Please briefly describe your responsibilities in your position as…….. (*Position listed above*).
2. Please describe which cervical cancer screening and treatment services that are available at this facility.
3. *Probe*: Document any of these if mentioned and probe for an exhaustive list of services: (*HPV screening/VIA screen/Pap Smear/Cryotherapy/LLETZ/ Biopsy, etc*.)
4. How often are these services provided in the facility?
   1. *Probe:* Are there certain dedicated days of the week?
5. Do you participate in cervical cancer prevention and control activities outside this facility? If yes, elaborate.

**HPV Screening**

1. Which method of sample collection for HPV testing is mostly offered at this facility: Clinician collection or self-collection by women? Why is it so? How is the choice or decision made on what is offered? Explain.
   1. Are all the service providers working in areas where HPV testing is done trained on cervical cancer screening? If not, what proportion are trained? e.g. 2 out of 4.

(Note: ***PROCEED to ASK questions related to Self-collection only IF it’s offered at the facility***]

- 1. *Probe*: If both sample collection approaches are offered, which of the two is more preferred by the women? Why do you think this is the case?

1. *Probe*: How do women receive their test results? How long does it take on average from sample collection to a women receiving the test results? (*look out for differences between the two methods of sample collection*).
2. How has the introduction of the HPV testing through self-collection of samples by women influenced how cervical cancer screening and treatment services availability and the volume and utilization of these services at this facility?
   1. *Probe:* If there has been a change, what do you think is the cause?
3. Has this facility been providing VIA or pap smear before HPV self-collection was introduced? How do you compare self-collection to other screening modalities in terms of time, resources, and of results for the woman?
4. How has the introduction of HPV self-collection of samples by women affected other health services provided in this facility?
   1. *Probe:* Has it had an impact on certain providers’ workload and availability to perform other duties? If yes, how? And laboratory testing?

**HPV Screening Acceptability**

1. How have women perceived HPV screening and VAT? Is it acceptable to them? Please describe why or why not.
   1. *Probe:* What reasons have woman mentioned for not accepting HPV screening?
   2. *Probe:* What health system-level barriers do you think might prevent a woman from doing HPV screening?
2. Are service providers and staff at your facility consider HPV screening with VAT a good option for cervical cancer prevention services? Please describe why or why not.

**Recommendations/Improvements**

1. Are there any improvements that could be made to HPV screening and VAT? If Yes, please describe regarding for the process of:

[*Prompt the respondent on the following where applicable*]:

- 1. Distributing HPV self-collection kits.
  2. Collecting HPV self-collection kits and transporting them to the lab
  3. Getting the results from the lab.
  4. Getting the results to the woman.
  5. Following up HPV-positive women to have VAT and treatment

1. How has integrating HPV screening with other services worked out in this facility?
   1. *Probe:* What are other implementation considerations, such as within the community?

**Scale up of HPV testing**

**[Interviewer reads:** SUCCESS project is supporting the MOH to make HPV testing and treatment with thermal ablation available to eligible women who need these services at this health facility and about 30 others in this country]

1. Regarding the MOH’s ability to support cervical cancer prevention and treatment:
   1. What are some enablers that you see to the adoption and scale up of HPV screening? Why do you say so?
   2. What are some potential barriers that you see to the adoption and scale up of HPV testing? Why do you say so?
2. Is the model of care or service delivery on cervical cancer currently in place in this facility scalable to other facilities offering the same level of care? Please explain why or why not.
3. Can you share any specific features that should be considered when designing community-based HPV screening?

**Wrap-up**

1. If an In-Charge from another facility asked your advice about how to implement these services in another facility, what lessons learned would you share?
2. Is there anything else you would like to tell me that you did not mention previously?

***thank the Facility or unit in-charge for his/her time and participation in the interview.***
