# Supplementary material for: Understanding integrated HPV testing and treatment of pre-cancerous cervical cancer in Burkina Faso, Cote d’Ivoire, Guatemala and Philippines: study protocol
Source: Reprod Health. 2023 Nov 13;20:167. doi: 10.1186/s12978-023-01696-8 (PMC10644460; doi:10.1186/s12978-023-01696-8)
Supplement: Supplementary file 1 — Additional file 1. Qualitataive data collection tools. [file 12978_2023_1696_MOESM1_ESM.zip › Qualitative tools/8-Key Informant Interview - Community Mobilizers.docx]

**Study Title:**  Feasibility and Acceptability of HPV testing and Treatment of Precancerous Cervical Lesions in Burkina Faso, Côte d'Ivoire, Guatemala, and Philippines

**Principal Investigator:** Mark Kabue, Dr.PH

**JHSPH IRB No.:** 13630

**PI Version/Date:** v2/ October 15, 2021

| **Data Collector Number:** |  |
| --- | --- |
| **Interview date:** |  |
| **Participant Study ID:** |  |
| **Number of years of experience as a Community Mobilizer:** |  |

***Instructions***

*Please use this form to interview Community Mobilizers. This interview is designed to gather information from Community Mobilizer about the integration of HPV screening and cervical cancer prevention and treatment messages in their work.*

*Before beginning the interview, please obtain informed consent from the respondent for their willingness to participate in the study and their permission to audio record the interview using the stamped consent form.*

**Introduction**

1. What is the title of your position?
2. Please describe your role in the community and the health system.
   1. *Probe:* Please tell me about a typical day for you in this role. How did you get involved in cervical cancer prevention and treatment services?

**Cervical cancer messaging**

1. Overall, what health education messages are you providing to women? What are women most concerned about?
   1. *Probe:* How feasible is it to include messages about cervical cancer prevention and HPV testing into the existing messaging platforms? If Yes, is it working well? Why or why not?
2. How comfortable are you explaining the causes of cervical cancer, how to prevent it, and HPV self-collection screening and treatment process to a woman (or women)? Explain your response.
3. Please describe if you are educating women about self-collection that they can do at a community health campaign.
4. Please describe how women in your community perceive HPV self-collection.
   1. *Probe:* Do they find HPV self-collection at a health facility more acceptable? What reasons do they cite?
   2. *Probe:* Do they find using HPV self-collection test kit at a community health campaign is acceptable? Has it been done in this community? If yes, describe what happened.
   3. *Probe:* Does it seem feasible for HPV self-collection to be offered to women to be do it in their own homes?
5. In general, what are some of the reasons that influence a woman to:
   1. Accept to do HPV self-collection at a community health campaign?
   2. Refuse HPV self-collection?
   3. Opt to have a clinician collect a HPV test sample from them at a health facility?
   4. Come for VIA screening if HPV-positive?
   5. Not return for VIA screening, if HPV-positive?
6. How do women get their lab test results at this facility, for example HIV, TB, blood sugar, etc.? How do they get the HPV test results?
   1. *Probe:* What is the best way for women to get their HPV test results? Go to the clinic? By phone? If SMS was an option?
   2. *Probe:* What are some of the challenges or concerns with getting HPV test results the ways described above?
7. As part of your job, what kind of IEC materials do you distribute to the women in your community? What’s the main source of the materials? Other sources?
8. Would it be easy to make this a routine part of your work? What would help you make this a routine part of your role and work in the community?
9. If a woman was given a test to do at home, at her convenience, how would the woman get that test back to the health facility?
   1. *Probe:* Would you be able to collect it later in the day or the next day?
   2. *Probe:* What are the transportation issues if she was going to bring it directly to the clinic?
10. What would be most helpful in encouraging or supporting women to do HPV self-collection at home or at the health facility?

**Treatment and follow-up**

1. From your perspective, what are the challenges or obstacles in educating women about importance of going for treatment if they are HPV positive? What can be done to make the messaging more effective?
2. What would be most helpful to encourage or support women HPV-positive to follow through with treatment?
3. What can be done to reduce the number of women who are lost to follow up after HPV screening? Share suggestions.
4. Can you share any specific features that should be considered when designing community-based HPV screening?
5. Is there anything else you would like to tell me that you did not mention previously?

***thank the COMMUNITY MOBILIZER for his/her time and participation in the interview.***
