# Supplementary material for: Understanding integrated HPV testing and treatment of pre-cancerous cervical cancer in Burkina Faso, Cote d’Ivoire, Guatemala and Philippines: study protocol
Source: Reprod Health. 2023 Nov 13;20:167. doi: 10.1186/s12978-023-01696-8 (PMC10644460; doi:10.1186/s12978-023-01696-8)
Supplement: Supplementary file 1 — Additional file 1. Qualitataive data collection tools. [file 12978_2023_1696_MOESM1_ESM.zip › Qualitative tools/9-Key Informant Interview - Laboratory personnel.docx]

**Study Title:**  Feasibility and acceptability of implementing integrated HPV testing and treatment of pre-cancerous cervical cancer lesions in Burkina Faso,  Côte d'Ivoire, Guatemala, and Philippines

**Principal Investigator:** Mark Kabue, Dr.PH

**JHSPH IRB No.:** 13630

**PI Version/Date:** v2/ September xx, 2021

| **Data Collector Number:** |  |
| --- | --- |
| **Interview date:** |  |
| **Participant Study ID:** |  |
| **Number of tests/ volume in month preceding the interview (disaggregated by type: e.g. HIV, TB, HPV, other):** |  |
| **Number of years of experience using each of these platforms:** | GeneXpert |
|  | Abbott |
|  | Roche/ cobas |
|  | Other (Specify) |

***Instructions***

*Please use this form to interview Laboratory Personnel. This interview is designed to gather information about service organization, laboratory testing, and the integration and sustainability of HPV screening at this facility.*

*Before beginning the interview, please obtain informed consent from the respondent for their willingness to participate in the study and their permission to audio record the interview using the stamped consent form.*

**Introduction:**

1. What is your job title?
2. How long have you been a ………………………. [***Job title mention in Qn 1***] at this facility?
3. Please describe your role at this facility.
   1. Probe: Please describe a typical work day; the different thing s/he does.
4. When were you trained in HPV testing?
   1. Which cervical testing platforms or machines are you familiar with?
      1. GeneXpert/ Abbott/ Roche/cobas or other platform? (*If mentioned, ask follow up questions related to the platform mentioned*]
      2. Other platform [*Do not ask questions on GeneXpert if not trained how to use it it*]

**Cervical Cancer related work**

1. How long have you been conducting cervical cancer testing or handling the samples at this facility and elsewhere?
   1. Probe: When do you provide these services at this facility? (e.g. days of the week)
   2. *Probe:* How many days per month?
2. What percentage of your day are you using the GeneXpert Machine processing cervical cancer samples?
   1. *Probe:* What are you processing?
   2. *Probe:* Do you have other responsibilities in addition to using the GeneXpert Machine to test the specimens you just described?
3. Describe the laboratory workload for this HPV testing.
   1. *Probe*: When does the workload increase and decrease? What are the reasons for this?
   2. *Probe*: If the workload flows, could your time be spent processing other samples/specimens/tests?
4. What are the benefits of having test machine capacity in the lab? What are the challenges on a day to day basis?
   1. *Probe*: What are your motivations for doing this work?
5. Please describe the courier system that transports HPV Self-collected samples to your laboratory.
   1. *Probe*: How many couriers deliver the samples to this laboratory?
   2. *Probe*: How often are samples delivered to your laboratory?
   3. *Probe*: Are the samples transported properly (e.g., at the correct temperature)?
   4. *Probe*: How could the process of transporting the samples to the laboratory be improved?
6. Please describe your experience in receiving and testing the specimen, reading and recording HPV test results, and then communicating these results with health facilities.
   1. *Probe:* Approximately how many days pass between the day you receive a specimen and the day you test the sample?
   2. *Probe:* Please describe the system that you use to record the results of the specimens.
   3. *Probe*: How do you communicate the results to the health facility?
   4. *Probe*: Approximately how many days pass between the day you test the sample and the day that you communicate the results to the health facility? Why does it take the number of days? What would it take to change the number of days between testing and sharing the results with the health facility?
   5. *Probe*: How could the process of sending results to the facilities be improved?

**Facilitators and Challenges**

1. What would make your work in processing the HPV results easier? Faster?
2. Please describe if you encounter problems with:
   1. Functioning of the test machine
   2. Stockouts or unavailability of supplies and consumables for running the HPV test
3. What do you think is the biggest challenge or obstacle throughout the process of receiving samples, testing samples, and communicating results to the health facility?
4. Given your experience with the study, please describe whether or not you think HPV self-collection is feasible. Please explain why or why not.
   1. *Probe:* How could processing the HPV self-collection samples be integrated with other types of testing, such as TB?
5. Please describe any reasons that you think MoHW or an individual facility might not want to process HPV samples on site.
   1. *Probe:* Please describe any reasons that you think MoHW or an individual facility might not want to offer HPV self-collection?
6. Please describe any improvements that could be made to the processing HPV Self-Collection results to make it more efficient.
   1. *Probe*: What would you tell another lab technician at another facility that may be thinking of processing the HPV samples?
   2. *Probe:* Can you share any specific features that should be considered when designing community-based HPV screening?
7. Is there anything else you would like to tell me that you did not mention previously?

***thank the Laboratory Personnel for his/her time and participation in the interview.***
