# Supplementary material for: Understanding integrated HPV testing and treatment of pre-cancerous cervical cancer in Burkina Faso, Cote d’Ivoire, Guatemala and Philippines: study protocol
Source: Reprod Health. 2023 Nov 13;20:167. doi: 10.1186/s12978-023-01696-8 (PMC10644460; doi:10.1186/s12978-023-01696-8)
Supplement: Supplementary file 2 — Additional file 2. Quantitative data collection tools. [file 12978_2023_1696_MOESM2_ESM.zip › Quantitative tools/1-Eligibility_Enrollment and Client Contact Form.docx]

**Study Title:**  Feasibility and acceptability of implementing integrated HPV testing and treatment of pre-cancerous cervical cancer lesions with thermal ablation in Burkina Faso,

Côte d'Ivoire, Guatemala, and Philippines

**Principal Investigator:** Mark Kabue, MPH, Dr.PH

**IRB No.:** 13630

**PI Version/Date:** v1/ May 19, 2021

**Overview: This form has 6 sections listed below as follows:**

SECTION 1: ELIGIBILITY ASSESSMENT CHECKLIST

SECTION 2: PREGNANCY and HIV RESULTS

SECTION 3: INFORMED CONSENT

SECTION 4: ENROLLMENT: DEMOGRAPHICS

SECTION 5: VISIT INFORMATION

SECTION 6: HPV RESULTS NOTIFICATION PREFERENCES AND CONTACT

**Brief study introduction script to be read to client by service provider.**

Hello, my name is ___________________[***Name of service provider***]. I am working with SUCCESS Project in collaboration with the ____________ [***Name of country’s***  ***Ministry of Health***] to carry out a study related to improving women’s health and cervical cancer prevention. This study has been approved by the ____________ [***Name of country’s Name of research ethics committee or board]***. The results will help the Ministry learn how best to offer cervical cancer prevention to women. We are offering a cervical cancer screening at this facility and it can be done today if you are interested.

***Instructions:*** Service provider READS a short script on Cervical cancer screening, explaining the purpose, benefits, and process, either clinician or self-collection of samples.

I will ask you some questions to find out if you qualify to take part. Is that okay?

YES -------- PROCEED

NO --------- END [Thank client and proceed with routine service delivery]

| *Health Provider Code / Name:* | ***[To be entered by Research assistant]*** |
| --- | --- |
| *Health Facility Code / Name:* | ***[To be entered by Research assistant]*** |

| *Participant Unique ID number:* | ***[Pre-filled in the screening form]*** |
| --- | --- |
| ^a^Clinic location in the facility where eligibility assessment, enrollment or HPV self-collection takes place:  ***Instructions****: Provider circles one location option* | Outpatient department 1  Family planning clinic 2  MCH clinic 3  HIV clinic 4  Other (specify) ________________ 5 |

| Date of Eligibility Assessment: |  |
| --- | --- |

***^a^Data collection devise (Phone/ Tablet) set to record GIS coordinates of location of where eligibility assessment is done.***

SECTION 1: ELIGIBILITY ASSESSMENT CHECKLIST

| **#** | **Question** | **Response/Codes** | | **Skip Patterns** |
| --- | --- | --- | --- | --- |
|  | Are you interested in finding out your HPV test results by using an HPV self-collection Kit or examination by a clinician? | YES . . . . . . . . . . . . . . . . . | 1 |  |
|  |  | NO . . . . . . . . . . . . . . . . . . | 0 | If **NO, STOP;** thank the respondent for her time. |
|  | *^b^*How old are you? [***If client does not know, probe for range (e.g., 18-24 or 25 – 49 years]*** | _______ years |  | **HIV Clinic:** <25 or above 49 years, **STOP**.  **Other Clinics**: <30 or above 49 years, **STOP.** |
|  | Do you normally seek health services in ---------------------  [***Name of Administrative Zone***]? | YES  NO | 1  0 | If **NO, STOP.** |
|  | Date of Last Menstrual Period | Date: |  | IF > 3 months |
|  | Are you currently pregnant? | YES  NO  Don’t Know | 1  0  98 | If **YES**, **STOP**.  If **NO or Don’t Know, Go to Qn 106** |
| 106 | Which family planning method are you currently using? | Pills  Condoms  Injectable (Depo)  Long Acting or Permanent Method  Natural Family Planning including rhythm or standard days  None  Abstinence  Other. Specify _____  Don’t know  Refused to answer | 1  2  3  4  5  6  7  8  9  97  98 | If LMP in Q104 is > 4 weeks and not using injectable, LARC or sterilization, then **perform pregnancy test (wait for Q201)**.  Note: if using injectable and last injection was more than 13 weeks/3 months, then **perform pregnancy test (wait for Q201)**  If using Injectable (and last injection was within last 13 weeks/3 months), LARC, sterilization method, then **SKIP to Q108.** |
|  | Have you given birth within the last 6 weeks? | YES  NO | 1  2 | If **YES, STOP**. |
|  | Have you had a hysterectomy?  *[****If client is not sure use PROBE*:** A hysterectomy is an operation that removes a part or all of your uterus/womb. It is different from having your “tubes tied.”] | YES  NO | 1  0 | If **YES, STOP**. |
|  | What is your HIV status? | Positive  Negative (must be documented and show result < 12 months old)  Never tested  Unknown / Negative result > 12 months  Refused to answer | 1  2  3  4  98 | If never tested, unknown,  undocumented negative test, or negative test >12 months, **finish eligibility questions and then refer for PITC and enter results in Section 2**. |
|  | Have you ever had a cervical cancer screening, for example by a PAP Smear, VIA or HPV Test? | YES  NO  Not Sure/Don’t know | 1  0  97 | If **NO, SKIP** to Q117  If **NOT SURE**, **SKIP** to Q113 |
|  | When was your last cervical cancer screening? | ____ Year _____ Month  Don’t know / Not Sure | 97 |  |
|  | What type of screening did you have in the past? | PAP Smear  VIA Screening  HPV Test  Don’t know/ Not sure | 1  2  3  97 |  |
|  | What was the result of the screening? | Positive / Abnormal  Negative / Normal  Suspected Cancer  Result not received  Don’t know / Not sure | 1  2  3  4  97 | If positive/ Abnormal, go to Qn 114.  If Negative / Normal, SKIP to Q117.  If suspected cancer, **STOP**. |
|  | For positive/abnormal results:  Did you receive treatment based on your screening results? | YES  NO | 1  0 | If **NO**, SKIP to Q116 |
|  | What kind of treatment did you receive for the positive or abnormal result? | Antibiotic  Cryotherapy  LEEP/LLETZ  Other treatment (specify) | 1  2  3  4 | If Cryotherapy, LEEP or Other Treatment provided, **STOP**. If antibiotic, Continue. |
|  | *For women who were positive or had an abnormal result but did not get treatment:*  Why did you not get treatment? | Told to rescreen  Treatment recommended but did not get treatment  Don’t know | 1  2  97 | Any response to this question, **STOP**. Client not eligible for the study but should be referred to VIA clinic. |
|  | A research assistant we will be asking for contact information so we can notify you of your HPV self-testing results. Study staff may contact you by phone up to three times and in-person up to two times to tell you your HPV test results. We would also contact you if you need referral or if you postpone a scheduled visit and miss the expected amount of time to return.  May we contact you either by phone or in-person? | YES  NO | 1  0 | If **NO, STOP. Client is NOT eligible for the study.** |
|  | Method of cervical cancer screening | Clinician sample collection  Self-sample collection | 1  2 |  |
| ***^b^Minimum age of enrolment is 25 years***  ***Instructions to provider***: [If the client has not done so already, ask them to get a pregnancy test (if directed in Q106) and an HIV test (if directed in Q109) and return with their results. Then proceed with Section 2.]  SECTION 2: PREGNANCY / HIV (PITC) RESULTS and ELIGIBILITY | | | | |
|  | *Result of pregnancy test (if needed from response to Q106)* | POSITIVE  NEGATIVE  PREGNANCY TEST NOT DONE or WOMAN REFUSED | 1  2  3  98 | If **POSITIVE** or if **PREGNANCY** **TEST NEEDED but Woman REFUSED**, **STOP**. |
|  | *Do you know your HIV status?*  *(If HIV testing done on the day of interview, use document the result)* | POSITIVE  NEGATIVE  INDETERMINANT | 1  2  3 | If enrolled in the other service areas, other than HIV clinic, proceed. |
|  | *Previous HIV screening history:*   - *Client has never screened before OR* - *Client does not know results of screening OR* - *Based on HIV test result, was the last cervical cancer screening more than 3 years ago for HIV positive client or more than 5 years ago for an HIV negative / unknown client?* | *(Pre-populated from answers earlier in this form)*  *YES*  *NO*  *Don’t Know / Not sure* | *1*  *0*  *3* | If enrolled in the other service areas, other than HIV clinic, proceed. For those enrolled in HIV clinic, the HIV status is already known (Positive) |
|  | Based on the previous answers, client is eligible or not eligible to take part in the study? | *(Pre-populated from answers earlier in this form)*  *ELIGIBLE. . . . . . . . . . . . .* | *1* | If ELIGIBLE, refer to the Data Collector to obtain informed consent. |
|  |  | *NOT ELIGIBLE . . . . . . . .* | *2* | If NOT ELIGIBLE, **STOP**. |

***Instructions to Research assistant***: Introduce yourself to the client and follow the and ask client for the paper filled by the clinician, then captures the information in an electronic devise (e.g. Tablet). If client is eligible for the study, **proceed to obtain** **informed consent**. Proceed to ask additional enrollment questions in sections 4-7, as per the skip patterns].

SECTION 3: INFORMED CONSENT CONFIRMATION

| Informed Consent obtained & documented: |  |
| --- | --- |

| **#** | **Question** | **Response/Codes** | | | **Skip Patterns** |
| --- | --- | --- | --- | --- | --- |
|  | *Informed consent to participate in obtained* | YES [**Tick Checkbox**] |  |  | |
|  |  | NO |  | If NO, **STOP** | |
|  | *Woman opted for cervical cancer through clinician collection of sample* ***[From Qn #118. ASK Qns in Section 7 in addition]*** | YES [**Tick Checkbox**]  N/A (If self-collection) |  | If YES, skip to Section 4  If N/A, go to 303 | |
|  | *Woman opted to use HPV self-collection of sample*  ***[From Qn #118]*** | YES [**Tick Checkbox**] |  | If YES or N/A, skip to Section 4 | |
|  |  | N/A |  |  |  |
|  | Would you be willing to answer a few questions related to why you are not interested in being screened for cervical cancer? (either self-collection or clinician collection of sample for testing). | YES . . . . . . . . . . . . . . . . .  NO . . . . . . . . . . . . . . . . . . | 1  0 | If YES, continue on to Section 4, 5, and 7.  If **NO, STOP**. | |

SECTION 4: DEMOGRAPHICS

**Interviewer**: I am now going to ask you some questions about yourself.

| **#** | **Question** | **Response/Codes** | | **Skip Patterns** |
| --- | --- | --- | --- | --- |
|  | Have you ever attended school? | YES . . . . . . . . . . . . . . . . . | 1 |  |
|  |  | NO . . . . . . . . . . . . . . . . . . | 0 | If **No**, skip to **Q403** |
|  | What is the highest level of school you attended: No school, primary, secondary or higher? | Primary  Secondary  Higher  No school | 1  2  3  4 |  |
| 1. 404 | What is your current marital status*?* | Married or living as married | 1 |  |
|  |  | Divorced/Separated  Widowed  Single or Never Married  Refused | 2  3  4  97 |  |
| 1. 405 | How many people are in your household including yourself? | _______ (Number only) |  |  |
| 1. 406 | What is your total family household income this year? *(****Read response choices for the country – Refer to data collector’s manual****)*? | Category 1  Category 2  Category 3  Category 4  Don’t Know  Refused to Answer | 1  2  3  4  97  98 | **Currency Units and brackets based on local currencies.** |
| 1. 407 | Prior to this visit, when was the last time you visited **this** facility*?* | Less than 1 month ago  1 – 6 months ago  6 – 12 months ago  More than 1 year ago  This is my first visit  Don’t know/ Can’t remember | 1  2  3  4  5  97 |  |

SECTION 5: VISIT INFORMATION

| **#** | **Question** | **Response/Codes** | | **Skip Patterns** |
| --- | --- | --- | --- | --- |
|  | What is the MAIN reason for coming to the facility today? | Family Planning services  Follow up visit for myself  Not feeling well  Follow up visit of family member  Family member not feeling well (Companion)  Cervical cancer screening  Other (specify)________ | 1  2  3  4  5  6  7 |  |
|  | About how long did it take for you to get to the clinic today? | Less than 20 minutes  20 – 60 minutes  Greater than 1 hour | 1  2  3 |  |
|  | Is this the closest facility to your residence that offers cervical cancer screening (any form)? | No  Yes | 0  2 |  |

SECTION 6: HPV RESULTS NOTIFICATION PREFERENCES AND CLIENT CONTACT DETAILS

***Instructions to be read to the client:*** A study clinician will contact you when the HPV test results are available. You have a choice in how you find out your results. The information will be kept confidential and delivered to you in a confidential manner. The questions I will ask next below will help you make the decision on how you receive your results.

| **#** | **Question** | **Response/Codes** | | |
| --- | --- | --- | --- | --- |
|  | What is your preference in terms of how you receive your test results? Would you like to be notified by phone or notified in person? | Notified by phone  Notified in person at the clinic by appointment (agreed upon time) | 1  2 | If notified in person, SKIP to Q607. |
|  | The clinician will inform you how you will return to the clinic to get my results. If we have to notify you of the test results via phone or SMS text, are you willing to tell us the phone number we can use? | YES  NO | 1  0 | If NO, skip to Q606 |
|  | *For women who agree to share a phone, ask:*  Do you share your phone with anyone else? | YES  NO | 1  0 |  |
|  | When we call you, we want to make sure we are actually reaching you and not someone else who has picked up your phone. Can you provide the name of your maternal grandmother’s first name or another code word that only you would know? |  |  |  |
|  |  | Example: Maternal Grandmother’s first name: ___________  …OR….  Code Word: __________ |  |  |
|  |  |  |  |  |
|  |  |  |  |  |
|  | If someone else answers the phone and you are not available, should we leave a message (“The Health Center called”) or not leave a message? | Leave a message  Do not leave a message  Leave the following message, “______” | 1  2  3 |  |
|  | If you miss your appointment or wish to receive your results by phone we will try to contact you by phone up to 3 times over a three-day period. After that we can send a community mobilizer to your home on 2 separate occasions to tell you that “your results are available at the clinic.”  Would you be willing to have a community mobilizer come to your home? | YES  NO | 1  0 |  |

|  |  |
| --- | --- |

| **Client Contact Details** | |
| --- | --- |
| Client’s Full Name (First name and Last or Family Name or Surname) | First: _____  Last or Family Name: ____  La |
| Cell phone number: |  |
| Can your phone receive text messages? | ______ YES or _____ NO |
| Alternate cell phone number: |  |
| Address or Place of residence (for in-person follow-up) (village, district, significant landmarks) |  |
| **Another point of contact:** | |
| Name |  |
| Relationship to client |  |
| Address (village, district) |  |
| Phone number (if available): |  |
| **Another Point of Contact:** | |
| Name |  |
| Relationship to client |  |
| Address  (village, district) |  |
| Phone number |  |

Thank the respondent.

END
