# Supplementary material for: Understanding integrated HPV testing and treatment of pre-cancerous cervical cancer in Burkina Faso, Cote d’Ivoire, Guatemala and Philippines: study protocol
Source: Reprod Health. 2023 Nov 13;20:167. doi: 10.1186/s12978-023-01696-8 (PMC10644460; doi:10.1186/s12978-023-01696-8)
Supplement: Supplementary file 2 — Additional file 2. Quantitative data collection tools. [file 12978_2023_1696_MOESM2_ESM.zip › Quantitative tools/2-HPV and Biopsy Results Form.docx]

**Study Title:** Feasibility and acceptability of implementing integrated HPV testing and treatment of pre-cancerous cervical cancer lesions with thermal ablation in Burkina Faso,  Côte d'Ivoire, Guatemala, and Philippines

**Principal Investigator:** Mark Kabue, Dr.PH

**JHSPH IRB No.:** 13630

**PI Version/Date:** v1/ May 19, 2021

| ***Instructions:*** *To be completed by lab staff at the time of the HPV testing specimen processing and for all histological requests for suspected cervical cancer cases (biopsy processing).* |
| --- |

| *Health Provider Code / Name:* |  |
| --- | --- |
| *Health Facility Code / Name:* |  |
| *Client Unique ID:* | *________________________________________________* |
| *Date of HPV Sample Collection:* | *(pre-populated at: HPV Sample Collection, otherwise enter date)* |
| *Date of reception of specimen by the lab* |  |
| *Date of HPV Test Specimen Processing (Results)* |  |
| *Date of reception of results by the health provider* |  |
| *Date of Biopsy Processing (Results)* |  |
|  |  |
| SECTION 1: Client Information | |

| **#** | **Question** | **Response/Codes** | **Skip Patterns** |
| --- | --- | --- | --- |
|  | *Client age* | *(pre-populated from Enrollment Form)* | |
|  | *HIV Status* | *(pre-populated from Enrollment Form)* | |

| SECTION 2: HPV Results |
| --- |

| **#** | **Question** | **Response/Codes** | | **Skip Patterns** |
| --- | --- | --- | --- | --- |
|  | *HPV test result* | Negative  Positive  Invalid, Error, No Result | 1  2  3 | If **POSITIVE**, Continue otherwise to end of form.  If Invalid, error or no result, client should re-test. |
|  | *HPV Test result 2 (for clients that had a invalid, error or no result on first HPV test result).* | Negative  Positive  Invalid, Error, No Result | 1  2  3 | If 2^nd^ retest results in invalid, error, no result, inform woman that test could not be completed and recommend to have VIA or pap smear performed. |
|  | *HPV Positive result details*  *(mark all that apply)* | HPV 16  HPV 18, 45  Other (Specify) | 1  2  3 |  |

| ***Instructions:*** *This section of the form should be completed by the clinician for all suspect cancer cases and LEEP cases that include a biopsy. Results should be obtained from IPMS.*  SECTION 3: Biopsy Results |
| --- |

| **#** | **Question** | **Response/Codes** | | **Skip Patterns** |
| --- | --- | --- | --- | --- |
|  | *Biopsy result* | Cancer  Not Cancer  Not determined | 1  2  3 |  |
|  | *Suspect Cancer Follow-up Management Plan* | Referred to PMH  Referred to another facility | 1  2 |  |
