# Supplementary material for: Understanding integrated HPV testing and treatment of pre-cancerous cervical cancer in Burkina Faso, Cote d’Ivoire, Guatemala and Philippines: study protocol
Source: Reprod Health. 2023 Nov 13;20:167. doi: 10.1186/s12978-023-01696-8 (PMC10644460; doi:10.1186/s12978-023-01696-8)
Supplement: Supplementary file 2 — Additional file 2. Quantitative data collection tools. [file 12978_2023_1696_MOESM2_ESM.zip › Quantitative tools/3-VAT Results and Treatment Form.docx]

**Study Title:**  Feasibility and acceptability of implementing integrated HPV testing and treatment of pre-cancerous cervical cancer lesions with thermal ablation in Burkina Faso,  Côte d'Ivoire, Guatemala, and Philippines

**Principal Investigator:** Mark Kabue, Dr.PH

**JHSPH IRB No.:** 13630

**PI Version/Date:** v1/ May 19, 2021

| ***Instructions:*** *This form should be completed by the provider/staff at the time of the visual inspection and treatment for all HPV positive clients.* |
| --- |

| *Health Provider Code / Name:* |  |
| --- | --- |
| *Health Facility Code / Name:* |  |
| Client Unique Identifier: | ________________________________________________ |
| ***Date*** *of VAT:* | *Time of VAT:* |
|  |  |
| SECTION 1: Client Information | |

| **#** | **Question** | **Response/Codes** | **Skip Patterns** |
| --- | --- | --- | --- |
|  | *Client age* | *(pre-populated from Enrollment Form)* | |
|  | *Date of Last Menstrual Period* | *Date: (pre-populated from Enrollment Form)* | |
|  | *HIV Status* | *(pre-populated from Enrollment Form)* | |
|  | *Screening and treatment history* | ***Screening Type****: New, VIA, Pap or DNA Test*  *(Some information is pre-populated from Enrollment Form)*  ***Date of last screening****: year of last screening (if NEW… blank)*  ***Result:*** *positive/abnormal or negative/normal or result not received (if NEW…. Blank)*  ***Treatment:*** *No, Cryotherapy, LLETZ, other (if NEW or negative… blank)* | |

|  |
| --- |
| SECTION 2: VAT and Treatment Results |

| **#** | **Question** | **Response/Codes** | | **Skip Patterns** |
| --- | --- | --- | --- | --- |
|  | *Was VAT completed today?* | YES  NO | 1  0 | If **YES**, **SKIP** to Q203. |
|  | *If VAT was not completed today, what was the reason?* | Severe Cervicitis / antibiotic given  Provider postponed  Other (specify______________) | 1  2  3 | If VAT not completed, STOP. Client is re-scheduled to first complete antibiotics before VAT is done. Provider can then enter the 2^nd^ / completed VAT into the Enrollment form. |
|  | *VAT Result* | Negative, eligible for Cryotherapy  Positive, eligible for Cryotherapy  Positive, large lesion – refer or treat with LLETZ  Suspect Cancer | 1  2  3  4 |  |

| **#** | **Question** | **Response/Codes** | | **Skip Patterns** |
| --- | --- | --- | --- | --- |
|  | *Was treatment performed today?* | YES, Thermal ablation treatment performed today  YES, cryotherapy treatment performed today  YES, LLETZ treatment performed today  NO, treatment postponed  NO, client referred for LLETZ  Client ineligible for treatment (suspect cancer) | 1  2  3  4  5  6 |  |
|  | *If no and eligible for thermal ablation or cryotherapy, reason why treatment not performed today?* | Client postponed  Provider postponed – equipment or supplies not available | 1  2 |  |
|  | *Date client will return for treatment* | Date: _____________ |  |  |
|  | *Other concerns/ notes* |  |  |  |
