# Supplementary material for: Understanding integrated HPV testing and treatment of pre-cancerous cervical cancer in Burkina Faso, Cote d’Ivoire, Guatemala and Philippines: study protocol
Source: Reprod Health. 2023 Nov 13;20:167. doi: 10.1186/s12978-023-01696-8 (PMC10644460; doi:10.1186/s12978-023-01696-8)
Supplement: Supplementary file 2 — Additional file 2. Quantitative data collection tools. [file 12978_2023_1696_MOESM2_ESM.zip › Quantitative tools/4-Referral Outcome Form.docx]

**Study Title:** Feasibility and acceptability of implementing integrated HPV testing and treatment of pre-cancerous cervical cancer lesions with thermal ablation in Burkina Faso,  Côte d'Ivoire, Guatemala, and Philippines

**Principal Investigator:**Mark Kabue, Dr.PH

**JHSPH IRB No.:**13630

**PI Version/Date:**v1/ May 19, 2021

| ***Instructions:*** *This form should be completed by the provider/staff at the time of receiving the HPV positive client that has been referred.* |
| --- |

| *Health Provider Code / Name:* |  |
| --- | --- |
| *Health Facility Code / Name:* |  |
| *Name of health facility receiving the referred client:* |  |
| *Client Unique Number:* | *________________________________________* |
| *Date of Referral:* |  |
|  |  |
| SECTION 1: Client Information | |

| **#** | **Question** | **Response/Codes** | **Skip Patterns** |
| --- | --- | --- | --- |
|  | *Client age* | *(pre-populated from Enrollment Form)* | |
|  | *Date of Last Menstrual Period* | *Date: (pre-populated from Enrollment Form)* | |
|  | *HIV Status* | *(pre-populated from Enrollment Form)* | |
|  | *HPV Result and Date* | *(pre-populated from HPV Result Form)* | |
|  | *VAT Result and Date* | *(pre-populated from VAT Result Form)* | |

|  |
| --- |
| SECTION 2: Referral Outcome |

| **#** | **Question** | **Response/Codes** | | **Skip Patterns** |
| --- | --- | --- | --- | --- |
|  | *Referral Diagnosis (reason)* | Large Lesion  Suspect Cancer  Other (specify_________) | 1  2  3 |  |
|  | *Was treatment procedure performed same day of referral?* | NO  YES - Thermal ablation  YES - Cryotherapy  YES - LEEP  YES – Biopsy taken | 0  1  2  3  4 | *If YES, Skip to Q204* |
|  | *If no, reason why procedure not performed today?* | Client postponed  Provider postponed – equipment or supplies not available  Other (specify________) | 1  2  3 |  |
|  | *Date client will return for further management if applicable (e.g., suspect cancer)* | Date: _____________ (otherwise leave Blank) |  |  |
