# Supplementary material for: Understanding integrated HPV testing and treatment of pre-cancerous cervical cancer in Burkina Faso, Cote d’Ivoire, Guatemala and Philippines: study protocol
Source: Reprod Health. 2023 Nov 13;20:167. doi: 10.1186/s12978-023-01696-8 (PMC10644460; doi:10.1186/s12978-023-01696-8)
Supplement: Supplementary file 2 — Additional file 2. Quantitative data collection tools. [file 12978_2023_1696_MOESM2_ESM.zip › Quantitative tools/5-Treatment Complication_Adverse Events Form.docx]

**Study Title:** Feasibility and acceptability of implementing integrated HPV testing and treatment of pre-cancerous cervical cancer lesions with thermal ablation in Burkina Faso,  Côte d'Ivoire, Guatemala, and Philippines

**Principal Investigator:**Mark Kabue, Dr.PH

**IRB No.:**13630

**PI Version/Date:**v1/ May 19, 2021

| ***Instructions:*** *This form should be completed by the provider at the time of the Treatment (cryotherapy, Thermal ablation or LLETZ) or at post-treatment follow-up, whenever the complication/adverse event encounter occurred.* |
| --- |

| Health Provider Code / Name: |  |
| --- | --- |
| Health Facility Code / Name: |  |
| Client Unique Number: |  |
| Date of Treatment: |  |
|  |  |
| SECTION 1: Client Information | |

| **#** | **Question** | **Response/Codes** | **Skip Patterns** |
| --- | --- | --- | --- |
|  | *Client age* | *(pre-populated from Enrollment Form)* | |
|  | *HIV Status* | *(pre-populated from Enrollment Form)* | |
|  | *Date of Treatment with (Cryotherapy / Thermal Ablation / LLETZ)* | *Date ________________* | |

| SECTION 2: Treatment/Post-treatment Complication |
| --- |

| **#** | **Question** | **Response/Codes** | | **Skip Patterns** |
| --- | --- | --- | --- | --- |
|  | *Treatment* | Thermal ablation  Cryotherapy  LLETZ | 1  2  3 |  |
|  | *Type of Complication* | Infection  Bleeding  Other (specify) | 1  2  3 | **If 3, notify PI for Adverse Event reporting.** |
|  | *Could the complication be managed using standard outpatient therapy (e.g., antibiotics, local coagulation)?* | Yes  No | 1  0 | If **YES**, and document in record to capture treatment complication rates.  If **NO, notify PI for Adverse Event reporting.** |
|  | *If the woman could not be managed with standard outpatient therapy, what management did she receive?* | - Hospitalization for antibiotic therapy - Hospitalization/packing for management of bleeding - Operating theater for management of bleeding - Other (specify) | 1  2  3  4 |  |
|  | *Outcome* | - Discharged/Full recovery - Discharged/prolonged recovery - Death | 1  2  3 |  |
