# Supplementary material for: Understanding integrated HPV testing and treatment of pre-cancerous cervical cancer in Burkina Faso, Cote d’Ivoire, Guatemala and Philippines: study protocol
Source: Reprod Health. 2023 Nov 13;20:167. doi: 10.1186/s12978-023-01696-8 (PMC10644460; doi:10.1186/s12978-023-01696-8)
Supplement: Supplementary file 2 — Additional file 2. Quantitative data collection tools. [file 12978_2023_1696_MOESM2_ESM.zip › Quantitative tools/6-Client Follow-Up Form.docx]

**Study Title:** Feasibility and acceptability of implementing integrated HPV testing and treatment of pre-cancerous cervical cancer lesions with thermal ablation in Burkina Faso,  Côte d'Ivoire, Guatemala, and Philippines

**Principal Investigator:** Mark Kabue, Dr.PH

**JHSPH IRB No.:** 13630

**PI Version/Date:** v1/ May 19, 2021

**Instructions**: The information on this form should be used each time a study staff person follows-up with clients during the study period, EXCEPT at 12 months AFTER treatment when a survey is conducted to ascertain the outcome of treatment.

| Client Unique Number: | ________________________________________________ |
| --- | --- |
| Study Staff Code: |  |
| Health Facility Name: |  |
| Date of Contact: |  |
| Time of Contact: |  |
| *If contacted via phone, retrieve SECRET code word recorded at Enrollment to verify identity (e.g. Name of maternal grandmother)* | *(pre-populate from Enrolment form: SECRET code word)* |

SECTION 1: Client Contact Attempts and Outcome

| **#** | **Question** | **Response/Codes** | |
| --- | --- | --- | --- |
| 1. | Reason for the client contact: | HPV test result available notification | 1 |
|  |  | Pathology result available notification  HPV positive: VAT past due  VAT completed: Cryotherapy past due  VAT completed: Thermal ablation past due  VAT completed: LLETZ past due  Other (Specify) | 2  3  4  5  6  7 |
| 2. | Method of client contact: | Phone Call | 1 |
|  |  | In-person contact with client in the community or at her home  Client presented at the clinic and contact made in person | 2  3 |
| *2a.* | *If in-person contact with client in the community or in her home, GIS coordinates where contact happened:* |  |  |
| 3. | Outcome of client contact | Client not reached and no message left for the client  Left message for client to come to the clinic  Client reached and client told to return to the clinic  HPV positive client reached and VAT appointment scheduled  HPV positive client reached and VAT scheduled after missing appointment | 1  2  3  4  5 |
|  |  | HPV positive client reached and treatment appointment scheduled after postponing cryotherapy or LLETZ  Client reached and appointment NOT scheduled because client refused appointment  Another outcome, specify (___________________________) | 6  7  8 |

Notes:

- *Log the number of contact attempts: #1, #2, #3, by reason for client contact, and type of client contact/ method of contact.*
- *Up to 3 attempts though phone or 2 by phone and 1 in-person per client. The attempts should be made on different day, at least one day apart, at different times of the day. All attempts should be made within a period of 3 weeks. It unsuccessful, record the client as “Lost to Follow up).*
- *Alert prompts for follow-up are generated based on time since previous contact and the next scheduled activity or procedure for the client.*
