# Supplementary material for: Understanding integrated HPV testing and treatment of pre-cancerous cervical cancer in Burkina Faso, Cote d’Ivoire, Guatemala and Philippines: study protocol
Source: Reprod Health. 2023 Nov 13;20:167. doi: 10.1186/s12978-023-01696-8 (PMC10644460; doi:10.1186/s12978-023-01696-8)
Supplement: Supplementary file 2 — Additional file 2. Quantitative data collection tools. [file 12978_2023_1696_MOESM2_ESM.zip › Quantitative tools/7-Twelve-month Post-treatment Follow-up Survey.docx]

**Study Title:** Feasibility and acceptability of implementing integrated HPV testing and treatment of pre-cancerous cervical cancer lesions in Burkina Faso,  Côte d'Ivoire, Guatemala, and Philippines

**Principal Investigator:** Mark Kabue, Dr.PH

**JHSPH IRB No.:** 13630

**PI Version/Date:** v1/ May 19, 2021

**Instructions**: The information on this form should ONLY during 12-month post-treatment survey.

| Client Unique Number: | _______________________ |
| --- | --- |
| Study Staff Code: |  |
| Health Facility Name: |  |
| Date of Contact: |  |
| Time of Contact: |  |
| # attempts:(*Circle the most appropriate*) | 1 2 3 |
| *If contacted via phone, retrieve SECRET code word recorded at Enrollment to verify identity (e.g. Name of maternal grandmother)* | *(pre-populate from Enrolment form: SECRET code word)* |

SECTION 1: Client Contact Attempts and Outcome

| **#** | **Question** | **Response/Codes** | | **Skips** |
| --- | --- | --- | --- | --- |
| 1. | *Type of treatment provided* ***(Information pre-populated from client’s data)*** | Cryotherapy  Thermal ablation  LLETZ | 1  2  3 | |
|  |  | Other (Specify) | 4 | |
| 2. | *Method of client contact:* | Phone Call | 1 Skip to Q3 | |
|  |  | In-person contact with client in the community or at her home  Client presented at the clinic and contact made in person | 2 Go to 2a  3 Skip to Q3 | |
| *2a.* | *Location of in-person contact with client if NOT at the health facility* | (Enter name of the place) |  | |
| 3. | Outcome of client contact | Client not reached and no message left for the client  Left message for client  Client reached and client told to return to the clinic for follow-up | 1  2  3 | |

Notes:

- *Log the number of contact attempts: #1, #2, #3, by reason for client contact, and type of client contact/ method of contact.*
- *Up to 3 attempts though phone or 2 by phone and 1 in-person per client. The attempts should be made on different day, at least one day apart, at different times of the day. All attempts should be made within a period of 3 weeks. It unsuccessful, record the client as “Lost to Follow up).*
- *Alert prompts for follow-up are generated based on time since previous contact and the next scheduled activity or procedure for the client.*

**SECTION 2: 12-Month post-treatment survey**

**Instructions**: *Data obtained from the clinician*

| **Date of Treatment:** | *(Pre-populated from Enrollment form)* |
| --- | --- |
| **Clinician assessment of client’s status 12-months after treatment** | Normal (No progression of disease) 1  Abnormal (Requires further evaluation) 2  Other (Specify) ___________________ 3 |
| **Client referred to another facility?** | YES 1  NO 0 |

***Instructions:*** *Research assistant asks client experience of care and health status during the 12-month review after receiving treatment.*

| **#** | **Question** | **Response/Codes** | | **Skip Patterns** |
| --- | --- | --- | --- | --- |
| *If contacted via phone, retrieve SECRET code word recorded at Enrollment to verify identity (e.g. Name of maternal grandmother)* | | *(pre-populate with grandmother’s first name or the code name already captured in the system)* | |  |
|  |  |  |  |  |
|  | **Question** | **Response / Codes** | | **Skip patterns** |
| 1 | How did your test results make you feel? | Upset  Worried/Concerned  Scared  Angry  Confused  Happy/Content  Other, specify_____  Don’t know  Refused to answer | 1  2  3  4  5  6  7  97  98 |  |
| 2 | How have you been after the treatment? | No problem  Experienced challenge (e.g. pain, etc) | 1  2 | Skip to Qn43 |
| 3 | What problem or challenge did you face after treatment?  ***[Mark all responses mentioned; DO NOT READ response***] | Minor aches and pains  Minor bleeding (did not go to health facility)  Serious bleeding (went to health facility)  Hospitalization for >1 day  Operated or treated at the same site again  Other (specify) | 1  2  3  4  5  6 |  |
| Please tell me how strongly you agree or disagree with the following statements. | | | | |
| 4 | Do you think visual assessment and treatment is safe? | Not at all  A little  A moderate amount  Completely  Don’t know  Refused to answer | 1  2  3  4  97  98 |  |
| 5 | Will you consider going for visual assessment and treatment in the future? | YES  NO  Don’t know  Refused to answer | 1  0  97  98 |  |
| 6 | Is there anything else you would like to tell us about your experience with cervical cancer prevention and treatment? | (**Free text)** | |  |
