# Supplementary material for: Understanding integrated HPV testing and treatment of pre-cancerous cervical cancer in Burkina Faso, Cote d’Ivoire, Guatemala and Philippines: study protocol
Source: Reprod Health. 2023 Nov 13;20:167. doi: 10.1186/s12978-023-01696-8 (PMC10644460; doi:10.1186/s12978-023-01696-8)
Supplement: Supplementary file 2 — Additional file 2. Quantitative data collection tools. [file 12978_2023_1696_MOESM2_ESM.zip › Quantitative tools/8-Client exit interview_with cost questions.docx]

**General Instructions:**

This questionnaire is designed to gather information related to cervical cancer cervices, from clients after receiving services from a health facility. The sections are: ***Demographics; Knowledge and awareness; HPV screening; Barriers and facilitators; Experience of care; and Cost of services.***

| **SUBJECT AREA** | **QUESTION/ITEM** | **Qn #** | **ANSWER CHOICES** | **ANSWER CODES** | **SKIP LOGIC** |
| --- | --- | --- | --- | --- | --- |
| **Facility information** | Participant Unique ID | **1** | *(pre-populate from Enrollment)* |  |  |
|  | Facility name (Code) | **2** | *(pre-populate from Enrollment)* |  |  |
|  | What service(s) did you come to the facility to receive today? [multiple response] | **3** | Family planning | **1** |  |
|  |  |  | HIV | **2** |  |
|  |  |  | Postnatal care | **3** |  |
|  |  |  | Cervical cancer-related | **4** |  |
|  |  |  | Other, Specify | **5** |  |
|  | About how far do you live from this health facility?  ***(Interviewer:Using your knowledge of the study area, guide the client to estimate the distance as closely possible)*** | **4** | ____________ (Kilometers) |  |  |
| 1. **Demographic characteristics** | | | | | |
| **Age** | How old were you during your last birthday? | **5** | ________ (years) |  |  |
| **Residence** | Where do you live?  Name of village/ locality________________  (***Interviewer: Use guide map provided to determine whether its rural or urban)*** | **6** | Urban area | 1 |  |
|  |  |  | Rural area | 2 |  |
| **Marital status** | What is your marital status? | **7** | Single | 1 |  |
|  |  |  | Married / Cohabiting | 2 |  |
|  |  |  | Widowed | 3 |  |
|  |  |  | Divorced/ Widowed | 4 |  |
|  |  |  | Refused to answer | 98 |  |
| **Religion** | What is your religion? | **8** | Roman catholic | 1 |  |
|  |  |  | Protestant | 2 |  |
|  |  |  | Muslim | 3 |  |
|  |  |  | Traditionalist | 4 |  |
|  |  |  | Other, Specify_______ | 5 |  |
|  |  |  | No religion | 6 |  |
|  |  |  | Refused to answer | 98 |  |
| **Education level** | What is your highest level of education? | **9** | Never attended school | 1 |  |
|  |  |  | Primary | 2 |  |
|  |  |  | Secondary or higher | 3 |  |
|  |  |  | Refused to answer | 98 |  |
| **Literacy level** | Can you read and write? | **10** | No, cannot read | 1 |  |
|  |  |  | Yes, can read with some difficulty | 2 |  |
|  |  |  | Yes, can read very well | 3 |  |
|  |  |  | No, cannot write | 4 |  |
|  |  |  | Yes, can write with some difficulty | 5 |  |
|  |  |  | Yes, can write very well | 6 |  |
|  |  |  | Refused to answer | 98 |  |
| **Occupation** | What is your current occupation or source of income? | **11** | Employed | 1 |  |
|  |  |  | Self-employment (self-employment) Small trades (informal) | 2 |  |
|  |  |  | Farming | 3 |  |
|  |  |  | Housewife | 4 |  |
|  |  |  | Unemployed (includes student) | 5 |  |
|  |  |  | Other (specify) | 6 |  |
|  |  |  | Refused to answer | 97 |  |
| 1. **Knowledge and Awareness** | | | | | |
| **Exposure to awareness activities** | Have you heard of human papillomavirus or HPV in the past year? | **12** | Yes | 1 |  |
|  |  |  | No | 0 |  |
|  |  |  | (Do not read) Don’t know | 97 |  |
|  |  |  | (Do not read) Refused to answer | 98 |  |
| **Exposure to awareness activities** | Have you heard information about cervical cancer in the past year? | **13** | Yes | 1 | If no, 17 |
|  |  |  | No | 0 |  |
|  |  |  | (Do not read) Don’t know | 97 |  |
|  |  |  | (Do not read) Refused to answer | 98 |  |
| **Exposure to awareness activities** | What did you hear about cervical cancer in the past year? Please share what you heard:   - Causes of cervical cancer - Risks for cervical cancer - How to prevent cervical cancer - Age range for women who should be screened for cervical cancer - Cervical cancer screening services available (probe for type of screening test—HPV or VIA) - Cervical cancer treatment services available (probe for type of treatment—thermal ablation, cryotherapy or LEEP/LLETZ) | **14** | ***Do NOT Read the possible response. Prompt with “What else?”***  **(Record all the responses mentioned)** |  |  |
| **Knowledge about cervical cancer screening** | Did you know anything about cervical cancer screening prior to your visit to the health facility today? | **15** | Yes | 1 |  |
|  |  |  | No | 0 |  |
|  |  |  | (Do not read) Don’t know | 97 |  |
|  |  |  | (Do not read) Refused to answer | 98 |  |
| **Exposure to awareness activities** | Do you recognize these materials (***show respondent up to 3 options as a visual prompt***)? | **16** | Yes | 1 |  |
|  |  |  | No | 0 |  |
|  |  |  | (Do not read) Don’t know | 97 |  |
|  |  |  | (Do not read) Refused to answer | 98 |  |
| **Exposure to awareness activities** | How have you heard about cervical cancer in the past year?  ***Do NOT Read the possible response. Prompt with “What else?”***  **(Record all the responses mentioned)** | **17** | Radio | 1 |  |
|  |  |  | TV | 2 |  |
|  |  |  | Campaign in my neighborhood/village | 3 |  |
|  |  |  | A friend/family told me about cervical cancer | 4 |  |
|  |  |  | Church | 5 |  |
|  |  |  | Facility Health worker | 6 |  |
|  |  |  | Community health worker | 7 |  |
|  |  |  | Health Facility/Post | 8 |  |
|  |  |  | School | 9 |  |
|  |  |  | Other, specify | 10 |  |
|  |  |  | (Do not read) Don’t know | 97 |  |
|  |  |  | (Do not read) Refused | 98 |  |
| **Exposure to awareness activities** | Who referred you for screening for cervical cancer? | **18** | CHW | 1 |  |
|  |  |  | Facility provider, came for other services | 2 |  |
|  |  |  | Self after hearing about availability of the screening | 3 |  |
|  |  |  | Other (specify) | 4 |  |
|  |  |  | (Do not read) Don’t know | 97 |  |
|  |  |  | (Do not read) Refused to answer | 98 |  |
| **Knowledge and Perceived Risks and Benefits** | How can cervical cancer be prevented? | **19** | Vaccination  Screening and treatment  Good personal hygiene  Other (specify) _________  Don’t know  Refused to answer | 1 |  |
|  |  |  |  | 2 |  |
|  |  |  |  | 3 |  |
|  |  |  |  | 4 |  |
|  |  |  |  | 97 |  |
|  |  |  |  | 98 |  |
|  | What do you believe causes cervical cancer? | **20** | HPV infection  Covid-19  Too many sexual intercourses  Too many sexual intercourses with different partners  Other (specify)_________  Refused to answer | 1  2  3  4  5  97 |  |
|  | Are HIV+ women at greater risk of developing cervical cancer than HIV- women? | **21** | Yes | 1 |  |
|  |  |  | No | 0 |  |
|  |  |  | (Do not read) Don’t know | 97 |  |
|  |  |  | (Do not read) Refused to answer | 98 |  |
|  | Do you know anyone who has been screened for cervical cancer? | **22** | Yes | 1 |  |
|  |  |  | No | 0 |  |
|  |  |  | (Do not read) Don’t know | 97 |  |
|  |  |  | (Do not read) Refused to answer | 98 |  |
|  | Please let me know if you agree, feel neutral (neither agree nor disagree), or disagree with **EACH** of the following statements:  **Perceived susceptibility**   - You may get cervical cancer some time during your life - It is likely that you will get cervical cancer in the future - Your chances of getting cervical cancer in the next few years are high   **Perceived severity**   - If you thought about cervical cancer you will worry - When you think about cervical cancer, you are afraid - Problems you would experience with cervical cancer would last a long time - Cervical cancer would threaten a relationship with husband, or partner - If you had cervical cancer your whole life would affected - If you developed cervical cancer, you would not live longer than 5 years - The risk of contracting COVID-19 outweigh the benefits of cancer treatment   **Perceived benefits**   - Having regular cervical cancer screening tests will help to detect changes to the cervix before they turn into cancer - regular screening tests are the best way to diagnose cervical cancer at an early stage - cervical cancer treatment would be tolerable/not so bad - Regular cervical cancer screening tests will decrease your chances of dying from cervical cancer - Having cervical cancer screening regularly will decrease worries about cervical cancer - Having cervical cancer screening will decrease your chances of getting infected by Covid-19 | **23** |  |  |  |
|  |  |  | Agree | 1 |  |
|  |  |  | Not sure/Neutral | 2 |  |
|  |  |  | Disagree | 3 |  |
|  |  |  | No answer | 98 |  |
|  |  |  |  |  |  |
|  | Where can you access cervical cancer screening services? | **24** | At this health facility | 1 |  |
|  |  |  | Other (specify): | 2 |  |
|  |  |  | (Do not read) Don’t know | 97 |  |
|  |  |  | (Do not read) Refused to answer | 98 |  |
| **C ) HPV Screening** | | | | | |
| **Type of Screening and acceptability of self-collection of sample** | Did you take the sample yourself or did a health worker take the sample?  “For an HPV test, a small stick or swab is put inside *the vagina to wipe the cervix, and the sample is sent to the laboratory to be tested. This can be done by a health-care provider or by a woman herself. When a woman does this process by herself, it is called self-collection of sample.”* | **25** | Yes, myself | 1 | Go 27  Go 26  Go to 30 |
|  |  |  | No, a health worker took the sample | 2 |  |
|  |  |  | Not screened for HPV | 3 |  |
|  |  |  | (Do not read) Refused to answer | 98 |  |
|  | Would you be willing to collect a sample by yourself to test for cervical cancer either at a health-care clinic, or in your home, if you were given instructions on how to collect the sample? **(For those who health worker collected sample):** | **26** | Yes | 1 |  |
|  |  |  | No | 0 |  |
|  |  |  | Don’t know / not sure | 97 |  |
|  |  |  | Refused to answer | 98 |  |
| **Feelings about screening** | How do you feel about your cervical cancer screening experience today? | **27** | Comfortabilitylikert scale with “0” being the lowest, and “7” the highest | 0-7 |  |
|  | Did the healthcare provider ask your permission/consent before performing the pelvic examination? | **28** | Yes | 1 |  |
|  |  |  | No | 0 |  |
|  |  |  | (Do not read) Don’t know | 97 |  |
|  |  |  | (Do not read) Refused to answer | 98 |  |
|  | Did you feel you had adequate privacy during the pelvic examination? | **29** | Comfortability likert scale with “0” being the lowest, and “7” the highest | [0-7] |  |
|  | Had you ever had a pelvic exam prior to your visit today? | **30** | Yes | 1 |  |
|  |  |  | No | 0 |  |
|  |  |  | (Do not read) Don’t know | 97 |  |
|  |  |  | (Do not read) Refused | 98 |  |
| **Attitudes and Norms about HPV screening/ pelvic exams** | How would you feel about having a pelvic examination in the future if it could help to prevent you from getting cervical cancer? | **31** | Comfortability likert scale  with “0” being the lowest, and “7” the highest | 0-7 |  |
| **Attitudes and Norms about HPV screening/ pelvic exams** | How do you think your women friends or relatives would feel about having a pelvic examination as part of a screening test if it could prevent them from getting cervical cancer? | **32** | Comfortability likert scale  with “0” being the lowest, and “7” the highest | 0-7 |  |
| **Attitudes and Norms about HPV screening/ pelvic exams** | How do you think your partner would you feel about you having a pelvic examination as part of a screening test if it could prevent you from getting cervical cancer? (***Only ask women who are either married or cohabiting***) | **33** | Comfortability likert scale  with “0” being the lowest, and “7” the highest | 0-7 |  |
| **Explanation by service provider** | How well did you understand the explanations given to you about cervical cancer screening and/or treatment services today? | **34** | Very well | 1 |  |
|  |  |  | Somewhat understood | 2 |  |
|  |  |  | Did not understand | 3 |  |
|  |  |  | (Do not read) Unsure | 97 |  |
|  |  |  | (Do not read) Refused | 98 |  |
| **Respectful care** | How would you gauge the length of time you spent today waiting to be served? | **35** | Very long wait time  Reasonable wait time  Don’t know / Not sure  Refused to answer | 1  2  97  98 |  |
|  | At any time during your visit to the facility today did you feel you were treated roughly physically by a health provider (e.g. slapped, pinched, or punched)? | **36** | Yes | 1 |  |
|  |  |  | No | 0 | If no, 41 |
|  |  |  | (Do not read) Don’t know | 97 |  |
|  |  |  | (Do not read) Refused to answer | 98 |  |
|  | At any time during your visit to the health facility today did you feel that the health workers talked to you in a rude or humiliating way (for example, shouted at, screamed at, insulted, scolded, or mocked you)? | **37** | Yes | 1 |  |
|  |  |  | No | 0 |  |
|  |  |  | (Do not read) Don’t know | 97 |  |
|  |  |  | (Do not read) Refused to answer | 98 |  |
|  | Did any health worker or other staff make negative comments to you regarding your sexual activity? | **38** | Yes | 1 |  |
|  |  |  | No | 0 |  |
|  |  |  | (Do not read) Don’t know | 97 |  |
|  |  |  | (Do not read) Refused to answer | 98 |  |
|  | Did any health worker or other staff sexually harass you or make sexual advances (for example, inappropriate touching or sexual comments that made you feel uncomfortable)? | **39** | Yes | 1 |  |
|  |  |  | No | 0 |  |
|  |  |  | (Do not read) Don’t know | 97 |  |
|  |  |  | (Do not read) Refused to answer | 98 |  |
|  | Did any health providers discuss your private health information in a way that others could hear? | **40** | Yes | 1 |  |
|  |  |  | No | 0 |  |
|  |  |  | (Do not read) Don’t know | 97 |  |
|  |  |  | (Do not read) Refused to answer | 98 |  |
|  | [*ask only if respondent said* ***“Yes”*** *to any abuse in Qns 36-40*]  Do you think that the poor treatment you described was influenced by any of the following?  (***Interviewer: Please read the list and circle any that apply*** ) | **41** | Your ethnicity /  language | 1 |  |
|  |  |  | Your religion | 2 |  |
|  |  |  | Your political beliefs or other beliefs | 3 |  |
|  |  |  | Poor/ Lack of money | 4 |  |
|  |  |  | My age  (very young) | 5 |  |
|  |  |  | Have many children | 6 |  |
|  |  |  | Marital status | 7 |  |
|  |  |  | Your gender | 8 |  |
|  |  |  | Level of education | 9 |  |
|  |  |  | Other (specify) | 10 |  |
|  |  |  | NA | 11 |  |
|  |  |  | (Do not read) Don’t know | 97 |  |
|  |  |  | (Do not read) Refused to answer | 98 |  |
| 1. **Barriers and Facilitators** | | | | | |
| **Filter question** | Have you been screened for cervical cancer and NOT received treatment before? | **42** | Yes  No  Not sure/ Don’t know  Refused to answer | 1 | Go to 43  Go to 44 |
|  |  |  |  | 0 |  |
|  |  |  |  | 97 |  |
|  |  |  |  | 98 |  |
| **Perceived Barriers to Treatment** | What is/are the reason(s) you did not receive treatment after the last HPV screening / testing?  Instructions: Do NOT Read the options, MARK all that are mentioned. | **43** | Was not told I needed treatment | 1 |  |
|  |  |  | Did not know how/where to get treatment | 2 |  |
|  |  |  | Too expensive for me | 3 |  |
|  |  |  | Clinic and/or laboratory are too far away | 4 |  |
|  |  |  | Poor service quality | 5 |  |
|  |  |  | Afraid of the procedure | 6 |  |
|  |  |  | Cultural beliefs do not allow | 7 |  |
|  |  |  | Afraid of Covid-19infection or other diseases - contamination | 8 |  |
|  |  |  | Family member would not allow it  (specify the relationship to the respondent _______________) | 9 |  |
|  |  |  | The facility where I used to go no longer offers treatment services | 10 |  |
|  |  |  | Other (specify): _____________ | 11 |  |
|  |  |  | **(Do not read)** Don’t Know | 97 |  |
|  |  |  | **(Do not read)** Refused to answer | 98 |  |
| **Perceived Barriers to Screening** | BEFORE today, if you have NOT been screened for cervical cancer, what is the is the MAIN reason for not doing so?  ***(Do NOT read responses; prompt the women to say the reason, then MARK all that are mentioned)*** | **44** | I did not know I needed to be screened | 1 |  |
|  |  |  | Afraid of the procedure or test results being positive | 2 |  |
|  |  |  | Did not know how/where to get the test done | 3 |  |
|  |  |  | Didn’t have time to go to a health facility have time | 4 |  |
|  |  |  | Health facility too far away | 5 |  |
|  |  |  | Poor services, e.g. rude service providers | 6 |  |
|  |  |  | Cultural beliefs do not allow me to be screened | 7 |  |
|  |  |  | Cannot afford to pay for the test | 8 |  |
|  |  |  | Health workers have not offered cervical cancer screening services to me | 9 |  |
|  |  |  | Afraid of Covid-19 (or other infectious diseases) contamination | 10 |  |
|  |  |  | Other (specify): ____________ | 11 |  |
|  |  |  | Don’t Know | 97 |  |
| **Perceived Facilitators to Screening** |  |  | Refused to answer | 98 |  |
|  | Please let me know if you strongly agree, agree, feel neutral **(agree, neither agree nor disagree, disagree)** with the following statements:   - You prefer a female doctor to conduct a cervical cancer screening - You would feel embarrassed to lie on a gynecologic examination bed and receive a pelvic exam - You are afraid to have a cervical cancer test for fear of a bad result - You neglect/forget to have a cervical cancer screening test regularly - if there is cervical cancer development in my destiny, having a screening test cannot prevent it - Having a cervical cancer screening test takes too much time - the cost of a cervical cancer test is high - Poor/disrespectful treatment by health workers is a concern for me - You have other problems more important than having a cervical cancer screening in your life - You are too old to have a cervical cancer screening regularly - There is no health facility close to your house to have a cervical cancer screening - Having a cervical cancer screening test is too painful - You are afraid of getting Covid-19 during the screening | **45** | Agree  Neutral / Not sure  Disagree  Refused to answer |  |  |
|  |  |  |  | 1 |  |
|  |  |  |  | 2 |  |
|  |  |  |  | 3 |  |
|  |  |  |  | 98 |  |
|  |  |  |  |  |  |
|  | What would make it easier for you to use cervical cancer screening services? **(Facilitators – Mark ALL that are mentioned)** | **46** | Having good understanding on why the test is important | 1 |  |
|  |  |  | Having more information on where to get the test | 2 |  |
|  |  |  | Less expensive to test | 3 |  |
|  |  |  | Health facility being near | 4 |  |
|  |  |  | Good quality of services at the facility, e.g respectful | 5 |  |
|  |  |  | Change of cultural beliefs | 6 |  |
|  |  |  | Change of opinion of family member | 7 |  |
|  |  |  | Reduced risk of exposure to covid-19 or other infection | 8 |  |
|  |  |  | Other (specify): _______________ | 9 |  |
|  |  |  | **(Do not read)** Don’t Know | 97 |  |
|  |  |  | **(Do not read)** Refused to answer | 98 |  |
| **Only if woman has a partner (married or cohabitating)** | You can initiate conversation about cervical cancer prevention services with your partner, he pays attention to what you have to say. | **47** | ***(Select on of the responses below for EACH of the statements on the Left side)*** | 1  2  3  97  98 | Response to Qn #7 MUST be « 2 », otherwise skip to Qn #48 |
|  |  |  | Agree Neutral/Not sure  Disagree (Do not read) Don’t know (Do not read) Refused |  |  |
|  | You can share your opinions about having a cervical cancer screening test (e.g., the HPV test) with your partner |  |  |  |  |
|  | You can share your opinions about being treated for cervical cancer (e.g., thermal ablation) with your partner |  |  |  |  |
|  | You can do a self-test for cervical cancer even if your partner does not want you to. |  |  |  |  |
|  | You can get screened for cervical cancer at a health facility even if your partner does not want you to. |  |  |  |  |
|  | You can get treated for cervical cancer at a health facility even if your partner does not want you to. |  |  |  |  |
|  | You have your partner's support to get screened for cervical cancer |  |  |  |  |
|  | You have your partner's support to get treatment for cervical cancer if needed |  |  |  |  |
| 1. **Experience of care** | | | | | |
| **Client satisfaction** | The services I received today were of good quality | **48** | strongly agree | 1 |  |
|  |  |  | agree | 2 |  |
|  |  |  | disagree | 3 |  |
|  |  |  | strongly disagree | 4 |  |
|  |  |  | **(Do not read)** Don’t know | 97 |  |
|  |  |  | **(Do not read)** Refused to answer | 98 |  |
| **Client satisfaction** | The services I received today met my needs | **49** | strongly agree | 1 |  |
|  |  |  | agree | 2 |  |
|  |  |  | disagree | 3 |  |
|  |  |  | strongly disagree | 4 |  |
|  |  |  | **(Do not read)** Don’t know | 97 |  |
|  |  |  | **(Do not read)** Refused to answer | 98 |  |
| **Client satisfaction** | If ever screened: Would you recommend cervical cancer screening at this facility to a friend or family member? | **50** | Yes | 1 |  |
|  |  |  | No | 0 |  |
|  |  |  | **(Do not read)** Don’t know | 97 |  |
|  |  |  | **(Do not read)** Refused to answer | 98 |  |
| **Perceptions of self-collection of sample (only for those who did self-collection for their most recent test)** | *Please indicate if you* ***agree, disagree****,* ***or neutral*** *with the following statements*: | **51** | Agree  Not sure/ Neutral  Disagree  Refused to answer | 1 2 3 98 |  |
|  | You felt embarrassed to take the sample yourself |  |  |  |  |
|  | You felt relaxed about taking the sample yourself |  |  |  |  |
|  | You were anxious during the self-collection |  |  |  |  |
|  | You were confident that the self-collection was performed correctly |  |  |  |  |
|  | You had difficulties doing the self-collection yourself |  |  |  |  |
|  | The explanation about the self-collection you were given seemed too complicated |  |  |  |  |
|  | You would prefer to have the sample taken by a health provider |  |  |  |  |
|  | You are willing to take the sample yourself next time |  |  |  |  |
|  | You are able to do the self-collection alone at home |  |  |  |  |
|  | You would recommend self-collection to those around you |  |  |  |  |
| **Perceptions of treatment (only for those who received treatment today)** | How would you rate any discomfort or pain you felt today during treatment to prevent cervical cancer on a scale of 0 to 7 0 means no pain, 7 that the pain is unbearable | **52** | Scale: 0 to 7 0 means no pain,7 that the pain is unbearable | 0-7 |  |
| 1. **Cost of services** | | | | | |
| **Out-of-pockets costs** | If you paid for transport to come here to the health facility today, how much did you pay? | **53** | Nothing |  |  |
|  |  |  | Amount: ___________ |  |  |
|  | How much time did it take for you to travel to the health facility? | **54** | Number of minutes |  |  |
|  | Did someone in your household come with you to the health facility today? | **55** | Yes | 1 |  |
|  |  |  | No | 0 |  |
|  | If yes, please mention relationship with that person | **56** | Open-ended |  |  |
|  | Did he/she lose any wages or money by coming with you? | **57** | Yes | 1 |  |
|  |  |  | No | 0 |  |
|  | Did the person that came with you pay for transport to get to the health facility? | **58** | Nothing  Amount:___________ |  |  |
|  | If you had to buy food and drink to come here, how much did you pay? | **59** | Nothing |  |  |
|  |  |  | Amount: ___________ |  |  |
|  | Did you lose any wages or any money by coming to the clinic? | **60** | Yes | 1 |  |
|  |  |  | No | 0 |  |
|  | How much money did you lose? | **61** | Amount:__________ |  |  |
|  | Did you have any other costs by coming here today that you wouldn’t have had normally? | **62** | Yes | 1 |  |
|  |  |  | No | 0 |  |
|  | What are they for and how much were they? | **63** | open-ended |  |  |
|  | Did you have to pay anything for the services you received today? | **64** | Yes | 1 |  |
|  |  |  | No | 0 |  |
|  | If yes, what did you pay for? How much did you pay? | **65** | (Open-ended) |  |  |
|  | How much time did you spend in waiting and attending the cervical cancer screening and/or treatment? | **66** | Number of minutes |  |  |
|  | Did your partner help you cover any costs by coming here today? | **67** | Yes Amount: ___________ |  |  |
|  |  |  | No |  |  |
|  | Did any health providers or other staff ask for a bribe or informal payment for better care? | **68** | Yes | 1 |  |
|  |  |  | No | 0 |  |
|  |  |  | (Do not read) Don’t know | 97 |  |
|  |  |  | (Do not read) Refused to answer | 98 |  |
|  | Did any health provider threaten not to take care of you because you could not pay or did not have required supplies ? | **69** | Yes | 1 |  |
|  |  |  | No | 0 |  |
|  |  |  | (Do not read) Don’t know | 97 |  |
|  |  |  | (Do not read) Refused to answer | 98 |  |
| **Recommend-ations** | Finally, do you have any suggestions which you think might improve the services at this facility? Please share them now. | **70** | **Open ended** |  |  |

**END**
